# Prediction of epigenetically regulated genes in breast cancer cell lines

**DOI:** 10.1186/1471-2105-11-305

**Published:** 2010-06-04

**Authors:** Leandro A Loss, Anguraj Sadanandam, Steffen Durinck, Shivani Nautiyal, Diane Flaucher, Victoria EH Carlton, Martin Moorhead, Yontao Lu, Joe W Gray, Malek Faham, Paul Spellman, Bahram Parvin

**Affiliations:** 1 Life Sciences Division, Lawrence Berkeley National Laboratory, Berkeley, CA, USA; 2 Affymetrix Inc., Santa Clara, CA, USA

## Abstract

**Background:**

Methylation of CpG islands within the DNA promoter regions is one mechanism that leads to aberrant gene expression in cancer. In particular, the abnormal methylation of CpG islands may silence associated genes. Therefore, using high-throughput microarrays to measure CpG island methylation will lead to better understanding of tumor pathobiology and progression, while revealing potentially new biomarkers. We have examined a recently developed high-throughput technology for measuring genome-wide methylation patterns called mTACL. Here, we propose a computational pipeline for integrating gene expression and CpG island methylation profles to identify epigenetically regulated genes for a panel of 45 breast cancer cell lines, which is widely used in the Integrative Cancer Biology Program (ICBP). The pipeline (i) reduces the dimensionality of the methylation data, (ii) associates the reduced methylation data with gene expression data, and (iii) ranks methylation-expression associations according to their epigenetic regulation. Dimensionality reduction is performed in two steps: (i) methylation sites are grouped across the genome to identify regions of interest, and (ii) methylation profles are clustered within each region. Associations between the clustered methylation and the gene expression data sets generate candidate matches within a fxed neighborhood around each gene. Finally, the methylation-expression associations are ranked through a logistic regression, and their significance is quantified through permutation analysis.

**Results:**

Our two-step dimensionality reduction compressed 90% of the original data, reducing 137,688 methylation sites to 14,505 clusters. Methylation-expression associations produced 18,312 correspondences, which were used to further analyze epigenetic regulation. Logistic regression was used to identify 58 genes from these correspondences that showed a statistically signifcant negative correlation between methylation profles and gene expression in the panel of breast cancer cell lines. Subnetwork enrichment of these genes has identifed 35 common regulators with 6 or more predicted markers. In addition to identifying epigenetically regulated genes, we show evidence of differentially expressed methylation patterns between the basal and luminal subtypes.

**Conclusions:**

Our results indicate that the proposed computational protocol is a viable platform for identifying epigenetically regulated genes. Our protocol has generated a list of predictors including COL1A2, TOP2A, TFF1, and VAV3, genes whose key roles in epigenetic regulation is documented in the literature. Subnetwork enrichment of these predicted markers further suggests that epigenetic regulation of individual genes occurs in a coordinated fashion and through common regulators.

## Background

### Epigenetic regulation and methylation-expression associations

Epigenetics refers to the study of heritable changes that cannot be explained by changes in the DNA sequence [1-4]. One mechanism of epigenetic regulation involves DNA methylation of CG dinucleotides, commonly represented as CpG. It is known that around 50% of the protein-coding genes are near CpG-rich sequences, known as CpG islands. Patterns of methylation in the CpG islands play an important role in regulating gene expression during both normal cellular development and disease processes. Increased methylation of CpG islands (hypermethylation) in tumor suppressor genes have been observed during tumor progression and metastasis as a result of aberrant methylation patterns [5,6]. At the same time, aberrations leading to decreased methylation of CpG islands (hypomethylation) of oncogenes are known to occur [7]. A review of epigenetics in cancer and the role of DNA methylation markers can be found in [8]. Since hyper and hypomethylation of the genome are considered widespread attributes of tumors, predicting the regulation of gene expression through CpG island methylation at an epigenome level will provide a better understanding of the tumor pathobiology and progression.

To measure genome-wide methylation, we used Target Amplification by Capture and Ligation (mTACL), a high-throughput technique developed by Affymetrix Inc., which has been used to measure the methylation of 145,148 CpGs in the promoters of 5,472 genes for 221 samples [9]. In the mTACL approach, regions of the genome to be analyzed (the targets) are first captured using dU probes. Such probes contain segments of DNA complementary to the targets with all the thymidines (T) substituted by uridines (U), and two common primers flanking the target sequences. mTACL has about 19,250 dU probes within the vicinity of transcriptional start sites of 5,472 genes, with 170,000 CpGs that are potentially relevant in tumorigenesis. Moreover, the dU probes were designed so that they hybridize specifically to target genomic DNA digested with restriction enzymes MspI and HpyF3I, along with adaptor oligonucleotides complementary to the common primers of dU probes. All cytosines (C) of the adaptor oligonucleotides were substituted with 5'-methyl cytosine (5-mC). dU probes, adaptor oligonucleotides and the target genomic regions were hybridized using the "touchdown annealing" protocol followed by ligation of oligonucleotides to the ends of the target genomic DNA. After ligation, the dU probes were removed by digestion using uracil DNA-glycosylase, leaving only the target genomic DNA ligated to common primers. Later, the target DNA was treated with bisulfite followed by amplification using common primers and hybridization to microarray containing 21-mer probes that span across the CpGs in the target DNA. The extent of CpG methylation is measured using relative signal of two probes (probsets) for each CpG: one corresponding to the case in which CpG(s) covered by the probe are methylated, and the other one to the sequence in which CpG(s) covered by the probe are unmethylated. There are at least 3 different probe sets that cover the same CpG. The resulting hybridization signals were translated into methylation values using logistic regression by fitting models of the relative probe signal to percentage methylation for each CpG. The regression used artificial samples of known CpG methylation (i.e. 0, 10, 25, 50, 75 and 100%) and the quality of fit was assessed with *r*^2^.

### Identifying epigenetically regulated genes

This paper discusses how a novel computational protocol can be used to integrate CpG methylation and gene expression data sets to systematically identify epigenetically regulated genes. Our assumption is that the effect of DNA methylation on gene expression is local and limited to the promoter region. A computational protocol on the exploratory analysis of epigenetic regulation using coupled methylation and expression data was proposed by Sjahputera *et al*. [10]. Their work investigated differential methylation hybridization and associated gene expression data to build a relational data space for non-Hodgkin's lymphoma. Fuzzy set theory is used to identify epigenetically regulated genes from the relational data space. In this process, methylation-expression associations were transformed into a logarithmic map, which was divided into four discriminative quadrants. Each quadrant represented one out of four gene regulation behaviors (i.e., hypermethylation and up-regulation; hypomethylation and up-regulation; hypermethylation and down-regulation; and hypomethylation and down-regulation). Clustering was applied to sets of associations, and the epigenetic regulation was determined from the cluster's location and quadrant's membership. A measurement of confidence is then computed from the probabilities involved in the determination of the clusters. This computational framework suffers from a number of limitations in the context of the high-dimensionality mTACL technology: (i) processing time of the high-volume relational data may be prohibitive; (ii) fuzzy clustering approaches are iterative and sensitive to the initial conditions, which may lead to unstable solutions; (iii) the division of quadrants is arbitrary and too rigid to incorporate the natural scale of data; and (iv) confidence in the solution is not established in terms of statistical significance (i.e., p-value).

To overcome the issues described above, we first reduced the dimensionality of the methylation data to alleviate the computational load resulting from the data. Consequently, this enables the efficient correlative analysis and assignment of p-values through permutation analysis that otherwise would be unmanageable in the original space. To this end, we used the following two-step clustering approach: (i) grouping along the genome to reveal regions with high concentration of assayed methylation sites, and (ii) clustering of methylation profiles within each region to identify similar methylation patterns. For the latter, we used spectral clustering, as it offers a number of advantages. For instance, it is noniterative; it can identify clusters along nonlinear boundaries; and it has been proven to outperform other techniques [11,12]. Its improved performance is attributed to the transformation of data into a higher-dimensional space, which requires less complex problem solving than in the original data space [13]. Here, a K-Spectral Clustering (KSC) is employed, and optimal input parameters are determined automatically. Secondly, associations between clustered methylation and gene expression data sets are produced by setting a fixed constraint of 20,000 base pairs in the vicinity of either 5' or 3' ends to match methylation sites to their genes. Finally, prediction and ranking of epigenetic regulated genes is performed based on logistic regressions of the methylation-expression associations onto an exponential curve. This logistic approach is flexible enough to incorporate any data scale and distribution, and does not contain rigid and arbitrary definitions that could limit its application. Finally, the significance of the logistic regression is verified by permutation analysis and computing the p-value.

### Computational protocol

**Figure 1:**
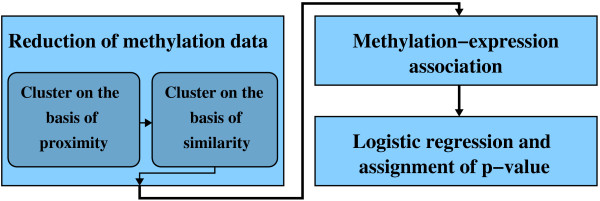
**Computational pipeline**. The computational pipeline for identification of epigenetically regulated genes from a panel of breast cancer cell lines. It was developed with the aim of (i) reducing the dimensionality of the methylation data, comprised of two sub-steps: (i.i) clustering of methylation profiles on the basis of proximity, and (i.ii) clustering within methylation sub-regions on the basis of similarity; (ii) associating the reduced methylation data to gene expression data; and (iii) ranking the methylation-expression associations according to their epigenetic regulation

The proposed computational protocol for identifying epigenetically regulated genes consists of three steps (Figure 1): (i) dimensionality reduction of the methylation profile, which is comprised of two sub-steps: (i.i) clustering of methylation profiles on the basis of proximity, and (i.ii) clustering within methylation sub-regions on the basis of similarity; (ii) association of the clustered methylation data to gene expression data; and (iii) logistic regression and ranking of the methylation-expression associations. Software and data can be downloaded at http://vision.lbl.gov/Software.

#### Dimensionality reduction

Genome-wide methylation measurements in CpG islands produce a high-volume data that make it computationally unmanageable for association, ranking, and required permutation analyses. Therefore, dimensionality reduction is a necessity and is implemented by us with two steps: (i) clustering on the basis of proximity of methylation sites within each chromosome along the genome; and (ii) clustering on the basis of similarity among methylation profiles across cell lines.

- Clustering on the basis of proximity: In this step, regions of concentration are identified by the proximity of CpG methylation sites along the genome. In each chromosome, methylation sites adjoining within 2,000 base pairs are aggregated and form distinct regions from methylation sites adjoined by more than 2,000 base pairs. Such regions provide a spatial context for methylation sites, grouping and isolating distant chromosomal regions. This is an important step for subsequent clustering based on the similarity of methylation profiles.

- Clustering on the basis of similarity: In this step, methylation profiles are clustered to identify cross-similarities within each region. Prior to the clustering, however, methylation profiles are pre-processed and represented by the largest principal components, which embed 99% of the data underlying variance. This is a standard approach and well documented in the machine learning literature. Clustering high-dimensional data in their principal component space results in lower computational complexity and lower risk from the *curse of dimensionality *[14]. The clustering method used here is unsupervised, and based on K-Spectral Clustering (KSC) [13], as discussed below.

Given a set of methylation profiles (*s*_*1*_,...*s*_*n*_) across *l *cell line principal components, the algorithm starts by computing an affinity matrix *A *whose diagonal elements *A*_*ii *_= 0 and off-diagonal elements are

Next, the *k *largest principal components are computed from the matrix

where *D *is a diagonal matrix whose *D*_*i,i *_elements are the sum of *A*'s *i*-th row. Let *X *be the *n *× *k *matrix that is formed by the *k *largest principal components of *L*. K-means clustering [15] is then applied to the normalized matrix *Y*, whose elements are represented by . Finally, the methylation profiles *S*_*i *_receive the same clustering assignment proposed for *Y *by k-means, i.e., a profile is assigned to cluster j if and only if row *Y*_*i *_is assigned to cluster *j*.

**Figure 2:**
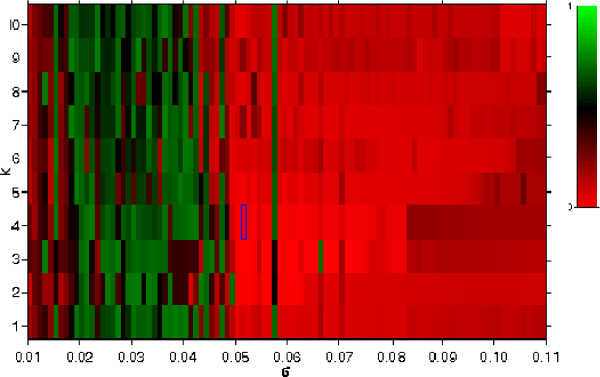
**Optimization example**. We tested our clustering approach on synthetic data with linear and non-linear boundaries to predict the validity of the results on real data. This example shows the determination of KSC's optimal parameters *σ *and *k *for the solution of a problem with samples distributed into three concentric circles. Each combination of *σ *and *k *produces a compactness value. The solution is selected from the set of parameters which produced the minimum value of compactness (marked by the blue box)

The above formulation of KSC requires parameter setting for the variables *σ *and *k*. *σ *determines the magnitude of the exponential decay in the computation of the affinity matrix A. Its value plays a role on the determination of boundaries between adjacent clusters. *k *specifies the number of clusters, which controls the amount of data quantization, but is often difficult to be determined in practice. A simple yet effective strategy to infer these parameters involves clustering with different combinations of the parameters and estimating the *compactness *of the inferred clusters. One way to characterize cluster compactness is to measure the cluster's internal homogeneity over external heterogeneity [16]. This relation can be mathematically defined by the ratio of , where *W *is the maximum distance between a point within a cluster and its center, and *B *is the minimum distance between two cluster centers. In our implementation, we partition the space of *k *and *σ *into fixed intervals, perform KSC for each enumerated pair of variables, and select the pair that produces the minimum measure of compactness. A representative methylation profile for each cluster is then computed by averaging all methylation measurements across cell lines. We tested this approach on synthetic data with linear and nonlinear boundaries to predict the validity of the results. Figure 2 demonstrates the selection of KSC's optimal parameters *σ *and *k *for synthetic data equally distributed into three concentric circles. The compactness measures, produced by each pair of parameters, are then normalized and shown as a heat map. The minimum value is marked by the blue box.

#### Methylation-Expression Association

Each representative methylation site, averaged over members of the same cluster, is associated with a gene or a set of genes. The association uses only the methylation site and the gene's probe set base range. A gene may have multiple probe sets in the expression data, which cover different portions of a chromosome. These associations are created for representative methylation sites being (i) within a gene probe set, or (ii) within a 20,000-base-pair window adjacent to the gene probe set. The latter accounts for natural uncertainties for locating a potential CpG island along the DNA.

#### Logistic regression and assignment of p-value

**Figure 3:**
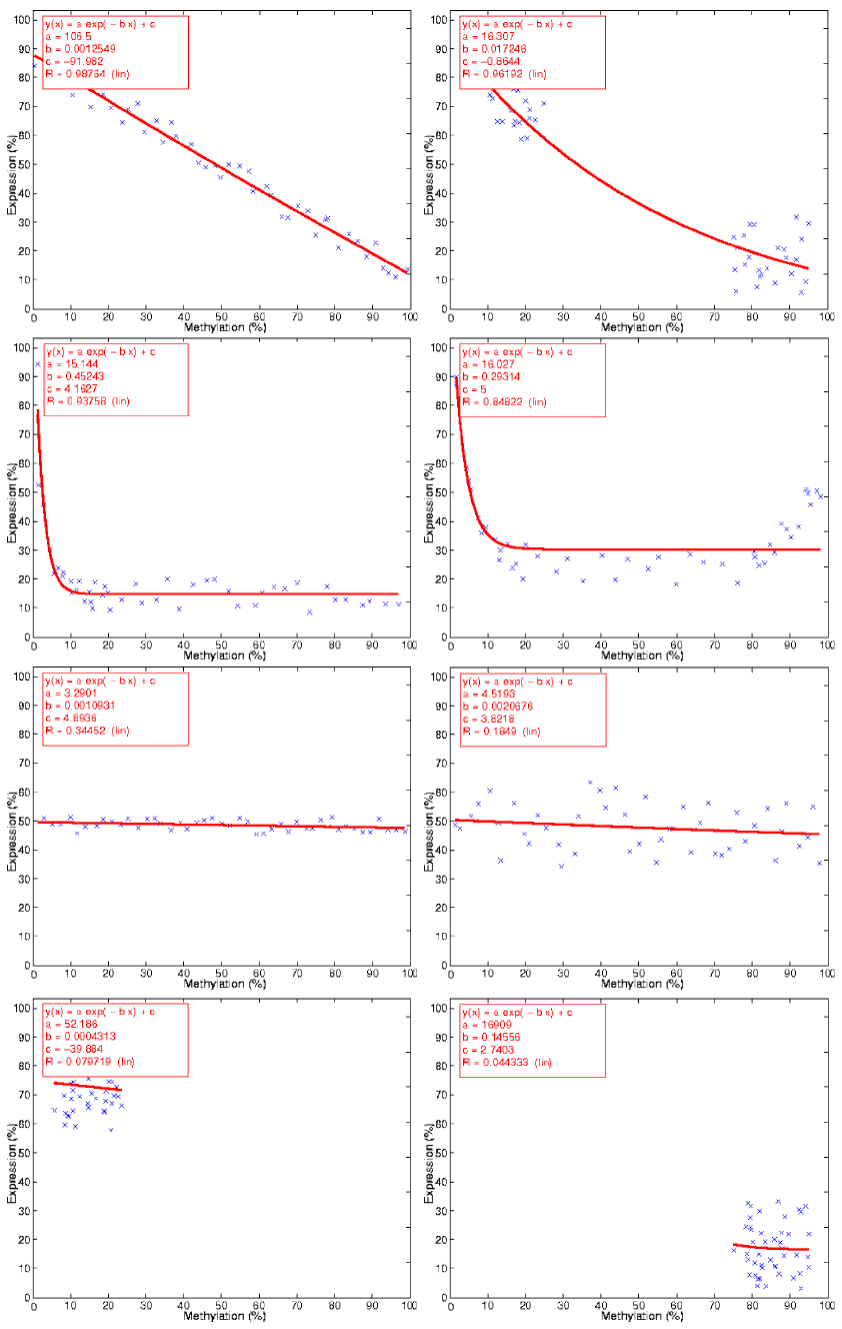
**Logistic regression**. Evaluation of the logistic regression on synthetic data reveals that expected inverse relationship between expression and methylation can be correctly ranked. *R *is the correlation coefficient and determines the quality of the logistic regression. The associations are ordered according to their *R *value and reflect the strength of the method's confidence on an epigenetic regulation. The logistic approach is flexible enough to incorporate any data scale and distribution, and does not contain rigid and arbitrary definitions that could limit its application

In order to characterize a negative correlation between the methylation and expression data (i.e., hypermethylation and down-regulation; and hypomethylation and up-regulation), we perform logistic regression of the methylation-expression associations. To this end, we experimented with several functional representations and propose a generic model, with flexible degrees of freedom, corresponding to an exponential curve of the form:

where *E *and *M *are respectively the expression and methylation measurements for each cell line, and a, b, and *c *are the free variables of the logistic model. Evaluation of the logistic regression on synthetic data reveals that expected inverse relationship between expression and methylation can be correctly ranked (Figure 3). Note that *R *is the correlation coefficient by which the associations are ranked. It reflects the quality of the logistic regression and, consequently, the method's confidence in an epigenetic regulation.

where *SSR *= ∑(*E*_*fit *_- *mean*(*E*))^2 ^and *SSE *= ∑(*E*_*fit *_- *E*)^2^, and *R *= 1 indicates a perfect fit to the model. The corresponding p-value is estimated for each association by computing:

The p-value is computed by comparing the value of *R *resulting from the curve regression, and the values of *R*_*m*_, *m *= 1, 2,...,*M*, resulting from *M *attempts for fitting the same curve after permuting the methylation measurements of each association. In our implementation, *M *is set at 10,000.

## Results and Discussion

### Data sets

The raw data was composed of 145,148 CpG methylation measurements containing, among others, the chromosome number, chromosomal sampling site, and the methylation profile across 58 cell lines. We filtered the original array to 137,688 CpG methylation sites, which contained valid chromosomal annotation data. With respect to gene expression data, we used publicly available data for a the panel of breast cancer cell lines [17]. Each array consists of 22,215 probe sets containing both luminal and basal subtypes. Forty-five cell lines were found in common between the ICBP expression data and the mTACL methylation data, and only data for the common cell lines were used for analysis (see Table 1). All expression measurements were transformed from the logarithmic scale to decimal units and normalized between 0-100% across the whole data set so the measurements were consistent with methylation.

**Table 1:** Panel of cell lines.

600MPE	AU565	BT20	BT474	BT483
BT549	CAMA1	DU4475	HBL100	HCC1143
HCC1187	HCC1428	HCC1500	HCC1569	HCC1599
HCC1937	HCC1954	HCC202	HCC2185	HCC38
HCC3153	HCC70	HS578T	LY2	MCF10A
MCF12A	MCF7	MDAMB157	MDAMB231	MDAMB361
MDAMB415	MDAMB435	MDAMB453	MDAMB468	SKBR3
SUM1315	SUM149PT	SUM159PT	SUM185PE	SUM44PE
SUM52PE	T47D	UACC812	ZR751	ZR75B

Forty-five cell lines were found in common between the ICBP expression and the mTACL methylation data. The cell lines listed here formed the gene signature used in our analysis.

### Dimensionality reduction

The first clustering step grouped the 137,688 methylation sites on the Affymetrix array into 5,785 distinct clusters (regions of concentration) across 23 chromosomes. Out of these 5,785 regions, the second clustering step generated 14,505 clusters, and produced representative methylation patterns by averaging the cluster's respective members. Note that this result represents a reduction of around 90% from the original raw data. Furthermore, 99% of the cell line's principal components' variance was found to be concentrated in 12 to 14 components, which reveals a high correlation between cell lines. Subsequent associations between the reduced methylation data and the gene expression generated 18,312 associations.

### Logistic regression and assignment of p-value

Table 2 shows the top 58 genes predicted as epigenetically regulated according to the logistic model and computed p-values. Methylation-expression associations for five well-known epigenetically regulated genes (i.e., collagen 1 *a*2 (COL1A2), trefoil factor 1 (TFF1), topoisomerase II*a *(TOP2A), cyclin-dependent kinase inhibitor 2A (CDKN2A), and vav 3 guanine nucleotide exchange factor (VAV3)) are also plotted on the right side for reference. One can note that the methylation patterns are highly heterogeneous for the panel of breast cancer cell lines.

**Table 2:** Gene ranking.

Gene	R	p-Value
COL1A2	0.888126	0.001200
S100A2	0.770036	0.008100
TFF1	0.764194	0.000000
INHBA	0.761402	0.000400
WNT5A	0.731727	0.002700
GJA1	0.722746	0.000300
GNG11	0.722025	0.000600
GSTM3	0.693819	0.000000
IGFBP5	0.684982	0.000000
IFI16	0.615905	0.002200
FDXR	0.611562	0.000500
CTGF	0.594878	0.000000
NUPR1	0.586186	0.000100
GSTP1	0.560942	0.004200
CYP1B1	0.550128	0.000200
TOP2A	0.522335	0.009000
ESR1	0.518515	0.015700
IFITM3	0.514558	0.002600
MX1	0.503719	0.012400
CDKN2A	0.500448	0.008800
CD44	0.496155	0.034700
MTHFD1	0.494175	0.028900
VAV3	0.481777	0.000800
TFAP2A	0.474620	0.000200
HOXA9	0.473453	0.000000
DHRS2	0.454703	0.009000
CBFA2T3	0.443504	0.021400
ZIC1	0.435035	0.016000
LITAF	0.434958	0.001700
ADAM12	0.428524	0.016000
IFITM2	0.421762	0.019400
EFS	0.412792	0.007300
TACSTD2	0.407764	0.006500
GSTO1	0.390240	0.010400
CGREF1	0.372320	0.000000
MAFB	0.366501	0.011300
CAMK2N1	0.353566	0.008600
SEMA3F	0.348895	0.000000
RAB25	0.347329	0.023900
ANXA13	0.341399	0.012600
ALCAM	0.335584	0.009400
EIF4B	0.328433	0.000000
GATA3	0.328377	0.008500
RAB21	0.321558	0.012700
PTN	0.320676	0.030900
PYCARD	0.319203	0.035600
MAPK13	0.316035	0.013700
IGFBP2	0.315176	0.021300
S100A6	0.310833	0.033000
C12orf24	0.310481	0.020100
IGFBP7	0.309320	0.049000
ALDH4A1	0.302697	0.000000
APITD1	0.296412	0.000000
CRABP2	0.285055	0.048900
ITGB4	0.281394	0.031500
BMP1	0.279983	0.001700
UNG	0.275001	0.000000
FAM134A	0.268202	0.043500

Top 58 genes predicted as epigenetically regulated according to our logistic model and p-value calculation.

**Figure 4:**
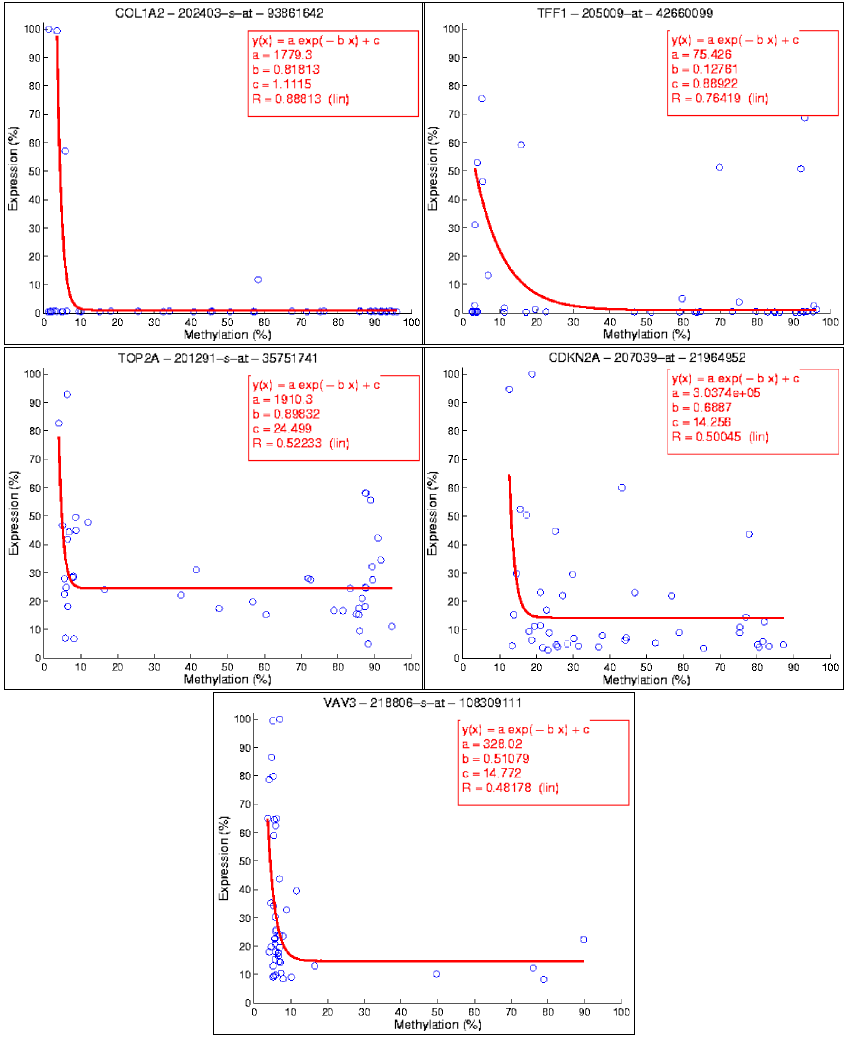
**Methylation-expression associations**. Five genes with known (based on the literature) epigenetic regulation demonstrate that logistic regression is appropriate as a model system. It is clear that the methylation patterns are highly heterogeneous for the panel of breast cancer cell lines

Methylation-expression associations for underlined genes are shown in Figure 4.

We have compared the percentage of selected markers with two cancer-specific gene data sets of (i) 5900 genes that The Cancer Genome Atlas Project (TCGA) is targeting for sequencing [18], and (ii) genes that were selected using Prediction Analysis of Microarrays (PAM) data as described in [17]. The TCGA gene set represents genes that are widely expressed in cancer whereas PAM gene set represents breast cancer subtypes. We found that 66% and 22% of our gene list are also in the TCGA and the PAM data sets, respectively. This analysis is promising since (i) the TCGA gene list is not specific to breast tissue, and (ii) the PAM data set does not incorporate methylation data; thus, by incorporating methylation data, a reduced number of biomarkers can be hypothesized.

### Epigenetically regulated genes

Our protocol has identified 58 genes that are epigenetically regulated. Here, we briefly discuss COL1A2, TOP2A, VAV3, CDKN2A, and TFF1 (genes underlined in Table 2 and respective methylation-expression associations shown in Figure 4), by comparing them against known literature. Methylation maps for 58 genes in relation to the regulated genes are shown in Additional file 1.

COL1A2 plays important role in collagen production and tumor development [19], and is hypermethylated and down-regulated in about 40% of the ICBP cell lines. Let us assume that hypermethylation and up-regulation accounts for measurements above the 50% threshold. It is interesting to note that our method has identified epigenetic regulation of COL1A2 even in the presence of only 3 up-regulated cell lines. These 3 lines are not outliers as the computational protocol has generated a hypothesis for further bioinformatics analysis. Epigenetic regulation of COL1A2 is consistent with the published literature, which suggests that its down-regulation correlates with hypermethylation, and is a frequent event in breast cancer cell lines such as MCF7 and HS578T [19]. Furthermore, aberrant methylation of COL1A2 has been identified in medulloblastoma and hepatoma [20,21], where biallelic methylation of COL1A2 was observed in 77% of medulloblastomas, in addition to be shown to distinguish histological subtypes of tumors [20]. TOP2A is an enzyme involved in controlling the topological state of the DNA machinery. Approximately 50% of ICBP cell lines are hypomethylated and up-regulated TOP2A. TOP2A is (i) a good prognostic marker in breast cancer and response to therapy [22], (ii) a prognostic factor for ER-positive breast cancer [23], and (iii) is epigenetically regulated for cellular assembly and organization in lymphoblastoid cell lines [24].

TFF1's function is not well known to date. However, it has been widely studied because of its presence in human tumors. For example, a recent study has identified and validated over-expression of TFF1 in breast carcinoma [25]. Another study has concluded decreased methylation levels in breast tumor cells [26], while a much older study states that TFF1 expression is regulated by DNA methylation in breast cancer [27]. VAV3 is a nucleotide exchange factor that activates rearrangement of actin filament, and its association shows that only 4 cell lines are hypermethylated. Epigenetic regulation of VAV3 is consistent with a recent report showing that 83% of breast tumors overexpress VAV3 [28].

CDKN2A is part of the cell cycle machinery and is an important tumor suppressor gene. Our analysis indicates that CDKN2A is hypermethylated and down-regulated in only about 30% of the samples, whereas the majority of the samples are hypomethylated and down-regulated. This discrepancy can be explained by DNA copy number loss or CDKN2A mutation, which is frequently associated with pathophysiology of certain types of cancers, including breast cancer [29-32].

### Subnetwork enrichment analysis

**Figure 5:**
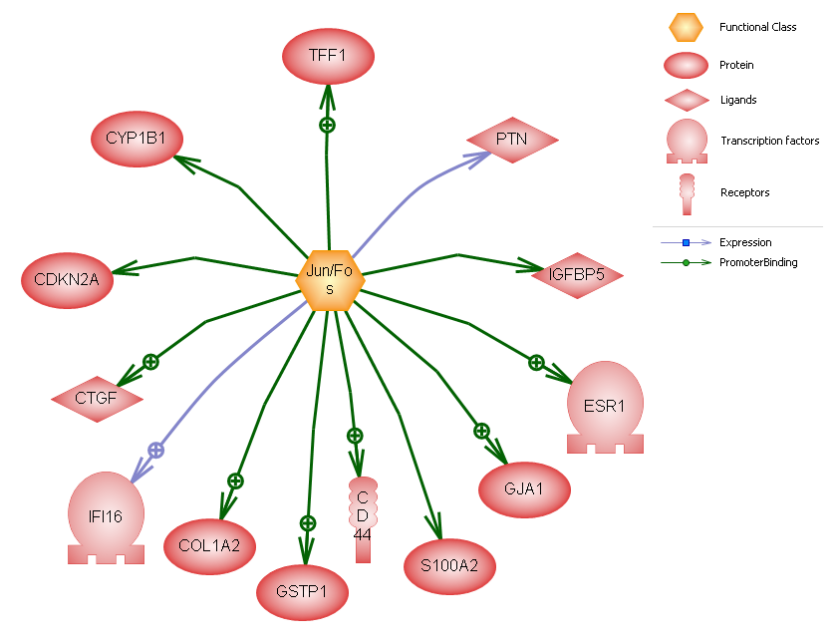
**Subnetwork enrichment (Jun/Fos).** Jun/Fos complex has been shown to be a common regulator for 13 of the predicted epigenetic biomarkers. Jun/Fos' subnetwork's statistical significance (p-value) is 1.68E-06, as shown in Table 3, row 2. Together with the AP1 transcription factor, Jun and Fos drive expression of a number of genes necessary for cell cycle progression

**Figure 6:**
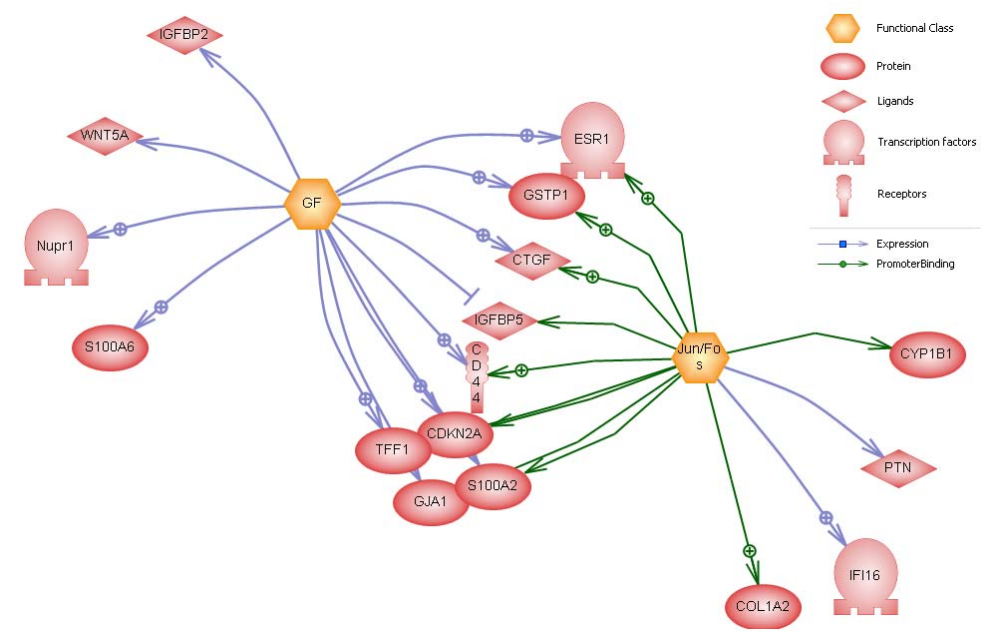
**Subnetwork enrichment (Jun/Fos and GP).** Interaction of two common regulators and their relations to 17 of the predicted epigenetic markers (Table 3, rows 1 and 2). Subnetwork enrichment and the presence of a large number of common regulators further substantiate our methodology

Although gene-by-gene analysis is a traditional and viable bioinformatics approach, modern analyses can benefit from enrichment strategies. Here, we have applied the predicted 58 genes for subnetwork enrichment through Pathway Studio. It is noteworthy that (i) 35 common regulators with 6 or more predicted genes have been identified, and (ii) that these regulators are of the type "Pathway". Table 3 lists the top 10 subnetworks according to their p-value. A complete spreadsheet of the common regulators and their targets can be found in Additional file 2. Analysis of these subnetworks suggests that epigenetic regulation of individual genes occurs in a coordinated fashion and through common regulators. An example is shown in Figure 5, where Jun/Fos complex has been shown to be a common regulator for a number of predicted epigenetic biomarkers. Jun and Fos, together with the AP1 transcription factor, drive expression of a number of genes necessary for cell cycle progression, including S100A2. S100A2 was also predicted by our protocol and has been implicated in breast cancer and its repression in tumor cells is mediated by site-specific methylation [33]. Figure 6 shows interaction of two common regulators and predicted epigenetic markers. Subnetwork enrichment and the presence of a large number of common regulators further substantiate our methodology.

**Table 3:** Subnetwork enrichment.

Common regulator	Predicted biomarkers	p-value
GF	S100A6,CD44,CDKN2A,CTGF,ESR1,IGFBP5,TFF1,GSTP1	2.86E-07
	IGFBP2,GJA1,WNT5A,NUPR1,S100A2	

Jun/Fos	CD44,CYP1B1,CDKN2A,CTGF,ESR1,IGFBP5,TFF1,GSTP1	1.68E-06
	GJA1,PTN,COL1A2,S100A2,IFI16	

EGF	CD44,CTGF,ESR1,IGFBP5,TFF1,KRT18,INHBA,IGFBP2	4.09E-06
	GJA1,PTN,COL1A2,S100A2	

TP53	CD44,CDKN2A,ESR1,SEMA3F,ANXA1,TFAP2A,COL1A2,LITAF	8.52E-06
	S100A2,TOP2A,FDXR,IFI16	

BMP2	CTGF,IGFBP5,INHBA,GJA1,WNT5A,CRABP2,COL1A2,ZIC1	8.56E-06

MAPK	GATA3,CD44,CDKN2A,CTGF,ESR1,IGFBP5,MAFB,TFF1	9.57E-06
	IGFBP2,GJA1,TFAP2A,COL1A2	

PKA	CYP1B1, CTGF, ESR1, IGFBP5, TFF1, KRT18, INHBA, IGFBP2	1.04E-05
	GJA1, TFAP2A	

TNF	S100A6,CD44,CYP1B1,CTGF,ESR1,IGFBP5,TFF1,GSTP1	1.71E-05
	INHBA,GJA1,PTN,IGFBP7,TFAP2A,COL1A2,MX1	

SRC	CD44,CTGF,ESR1,TFF1,KRT18,IGFBP2,COL1A2	2.30E-05

TGF family	GATA3,CD44,CDKN2A,CTGF,IGFBP5,INHBA,GJA1,IGFBP7	2.89E-05
	CRABP2,COL1A2,VAV3	

Top 10 lowest p-valued pathways identified by subnetwork enrichment analysis of the predicted 58 genes through Pathway Studio (see complete list in Additional file 2). Common regulator, genes involved and respective p-values are shown. The occurrence of multiple predicted markers within the same pathway suggests that epigenetic regulation of individual genes occurs in a coordinated fashion and through common regulators.

### Subtype-specific epigenetic regulation

**Figure 7:**
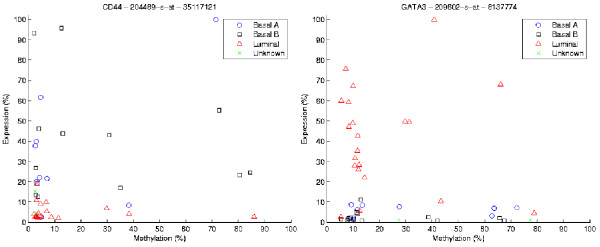
**Cell line subtypes.** Methylation-expression associations for CD44 (basal A and B specific gene) and GATA3 (luminal specific gene) according to the cellular subtype. There is evidence that the methylation pattern reflects the basal and luminal subtypes in breast cancer cell lines

One aspect of data analysis in cancer biology is to identify subtypes within the tumor lines. Our analysis indicates that there is evidence of subtype methylation with respect to the previous classification of basal A, basal B, and luminal lines, where these subtypes were shown to have DNA copy number changes similar to those of the respective subtypes found in primary breast tumors [17]. Our protocol suggests that the luminal marker GATA3 and basal marker CD44 are (i) epigenetically regulated and (ii) cell line specific. For example, Figure 7 indicates that luminal lines have low expression as a function of methylation for CD44 (basal A and B-specific genes), although the basal lines are epigenetically regulated; and (ii) the opposite holds for GATA3 (luminal-specific genes).

## Conclusions

In this paper, we proposed a computational pipeline for identifying epigenetically regulated genes for a panel of breast cancer cell lines. The protocol avoids excessive computational complexity through a step-wise reduction of methylation data for the required expression data associations. To this end, a twofold clustering approach explored both the proximity of methylation sites and similarities among methylation profiles across cell lines. K-Spectral Clustering was presented and used in the latter step. As a result of data clustering, a number of representative methylation profiles were generated for direct association with candidate genes. Epigenetic regulation was estimated from logistic regressions of the methylation-expression associations and its significance verified through the computed p-value. The computational pipeline was applied to a panel of 45 breast cancer cell lines, and the protocol identified a list of 58 genes, including COL1A2, TOP2A, TFF1, and VAV3, whose key roles in epigenetic regulation are consistent with known literature. Subnetwork enrichment of these markers identified 35 common regulators of the type "Pathway" with 6 or more predicted genes, further suggesting that epigenetic regulation of individual genes occurs in a coordinated fashion and through common regulators. Our current efforts focus on associating methylation data with the therapeutic responses and other biological data derived from the same panel of cell lines.

## Competing interests

The authors declare that they have no competing interests.

## Authors' contributions

LAL and BP designed and implemented the computational protocol, and drafted the manuscript. PS and JWG established the biological questions and computational requirements, designed the experiment and provided material to Affymetrix. AS contributed to some biological analysis of the results, assisting with suggestions to the manuscript. SD produced the maps of methylation for the epigenetically regulated genes. SN, DF, VC, MM, YL and MF conducted the experiments resulting in available data from the ICBP panel of breast cancer cell lines. All authors read and approved the final manuscript.

## Supplementary Material

Additional file 1 **Maps of methylation for the epigenetically regulated genes**. Each graph shows a map of methylation in relation to the top 58 epigenetically regulated genes from Table 2. The respective R values are represented by the magnitude of the yellow bars, plotted over the methylation site responsible for the highest ranked association with each gene Click here for file

Additional file 2 **List of common regulators through subnetwork enrichment**. Subnetwork enrichment analysis of the predicted 58 genes through Pathway Studio identified 35 common regulators of the type "Pathway" with 6 or more predicted genes. Common regulator, subnetwork type, total number of neighbors, number of overlapping markers, percentage of overlapping markers, gene set seed, overlapping markers, p-value and rank number are shown. Click here for file
