# Supplementary material for: Prediction of epigenetically regulated genes in breast cancer cell lines
Source: BMC Bioinformatics. 2010 Jun 4;11:305. doi: 10.1186/1471-2105-11-305 (PMC2903569; doi:10.1186/1471-2105-11-305)

COL1A2 logistic correlation index

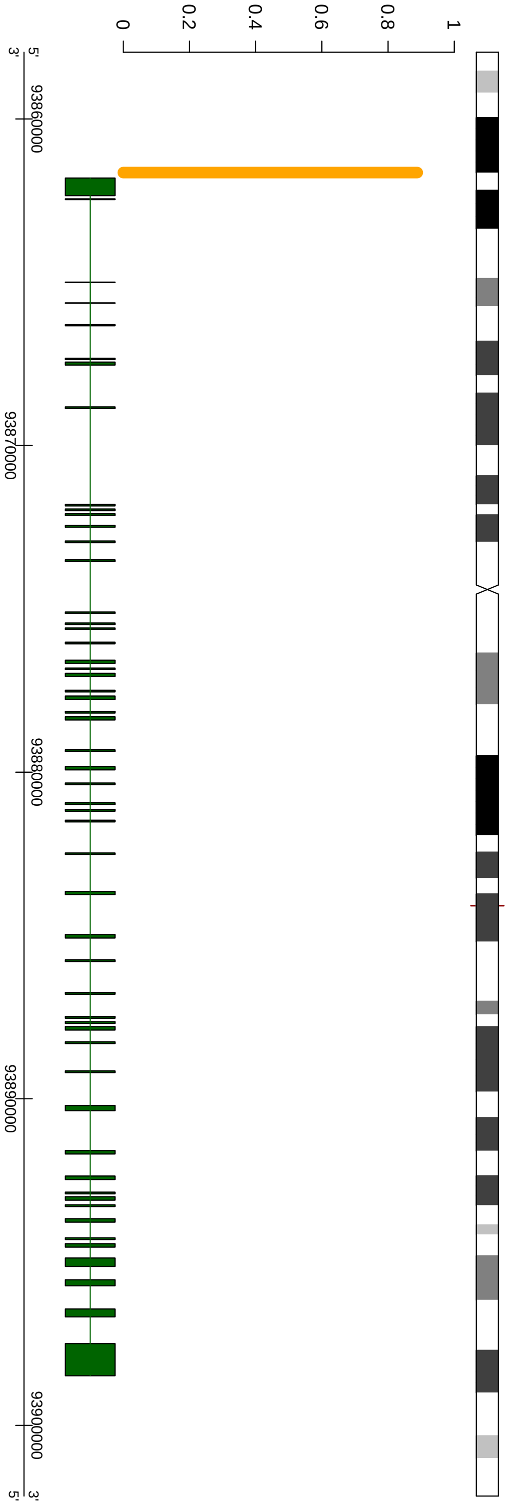

S100A2 logistic correlation index

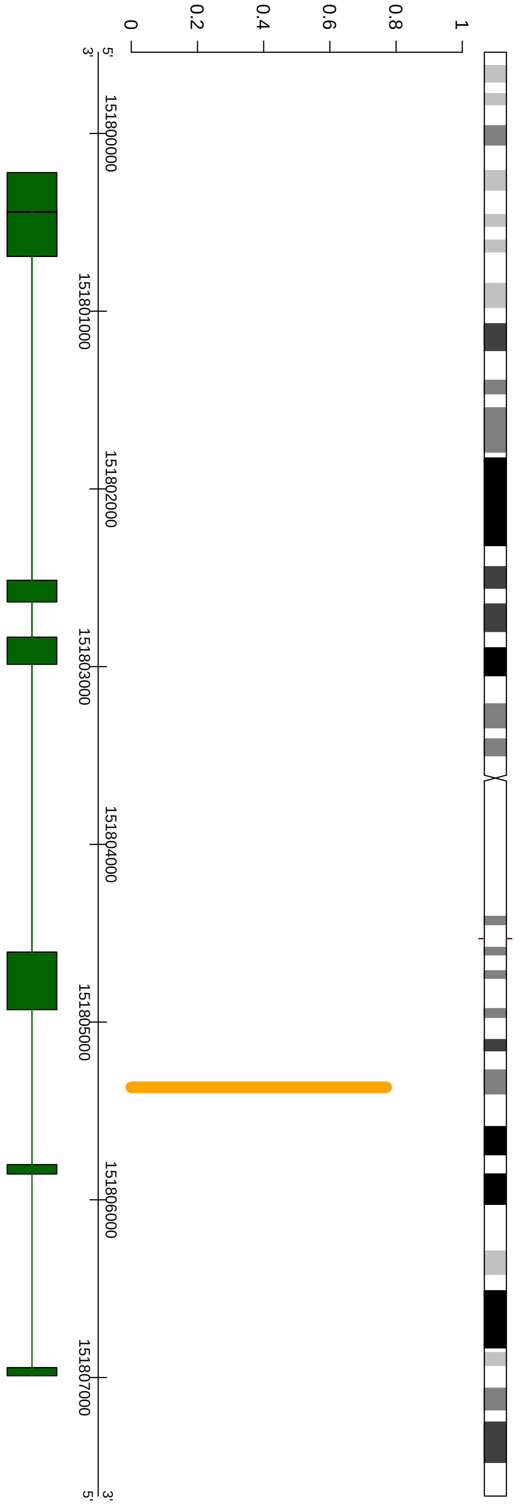

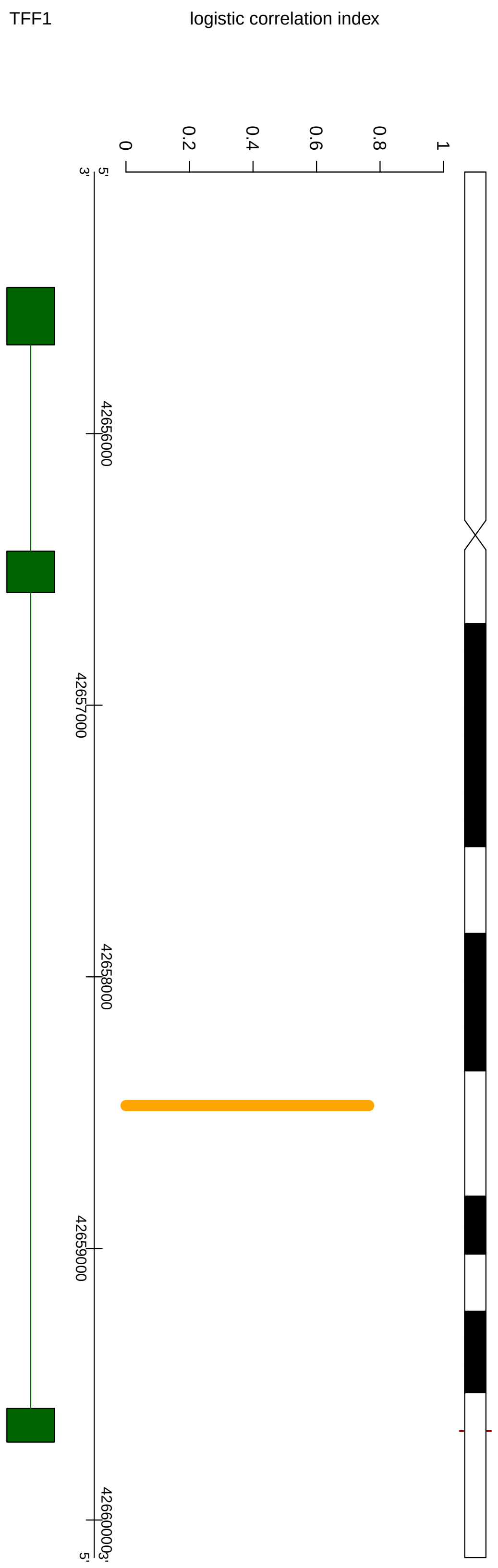
TFF1
logistic correlation index
0
0.2
0.4
0.6
0.8
1
5'
3'
42656000
42657000
42658000
42659000
42660000
3'
5'

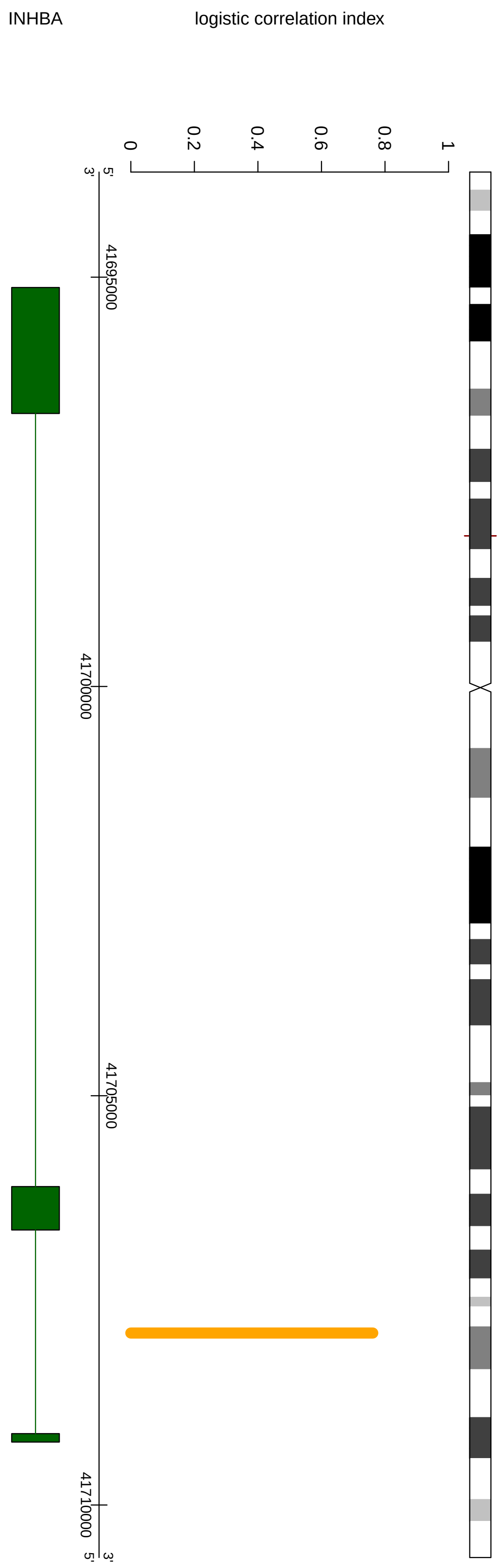
INHBA
logistic correlation index
0
0.2
0.4
0.6
0.8
1
5'
3'
41695000
41700000
41705000
41710000
3'
5'

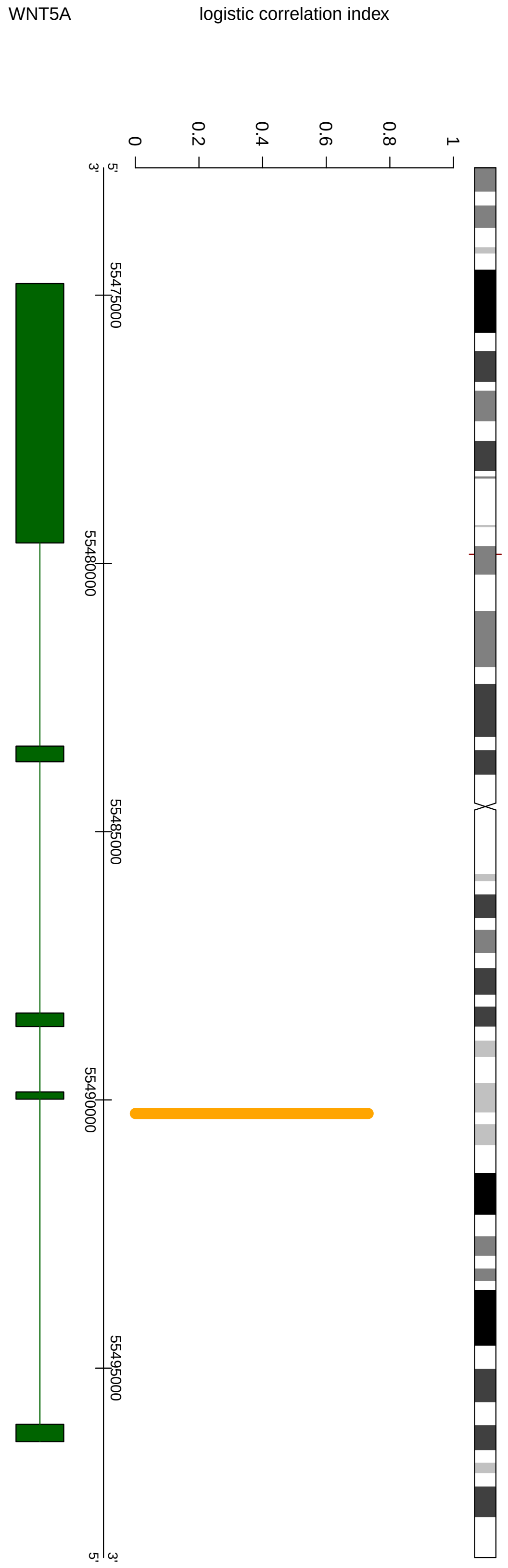
WNT5A
logistic correlation index
0
0.2
0.4
0.6
0.8
1
5'
3'
55475000
55480000
55485000
55490000
55495000

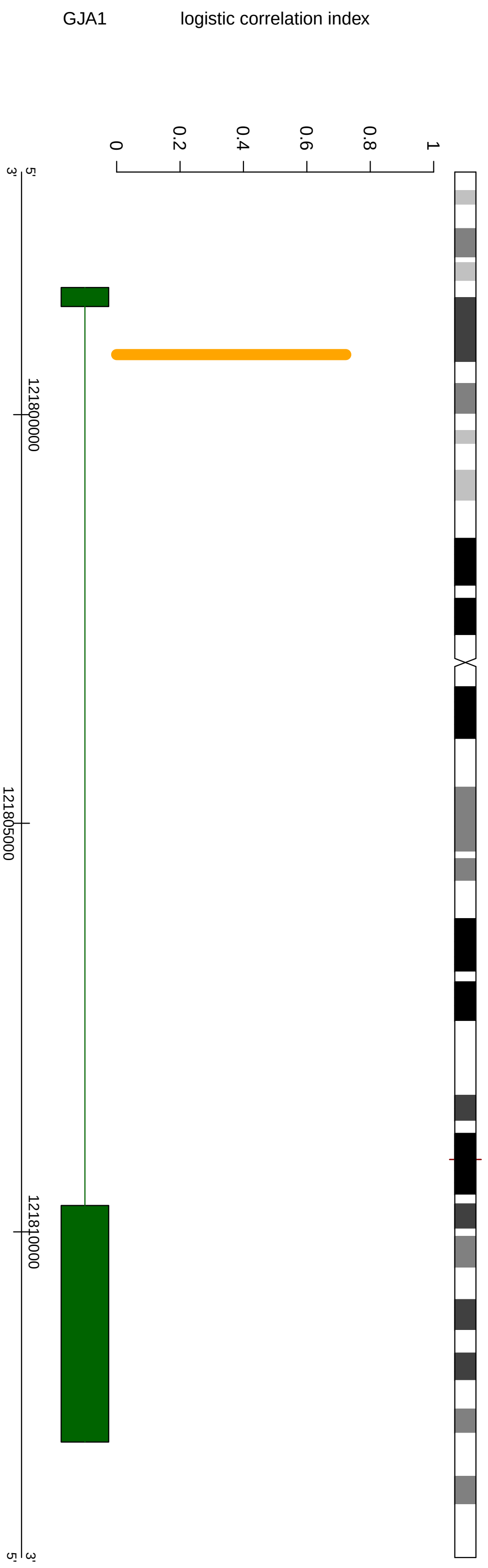
GJA1
logistic correlation index
0
0.2
0.4
0.6
0.8
1
121800000
121805000
121810000
5'
3'
3'
5'

GNG11 logistic correlation index

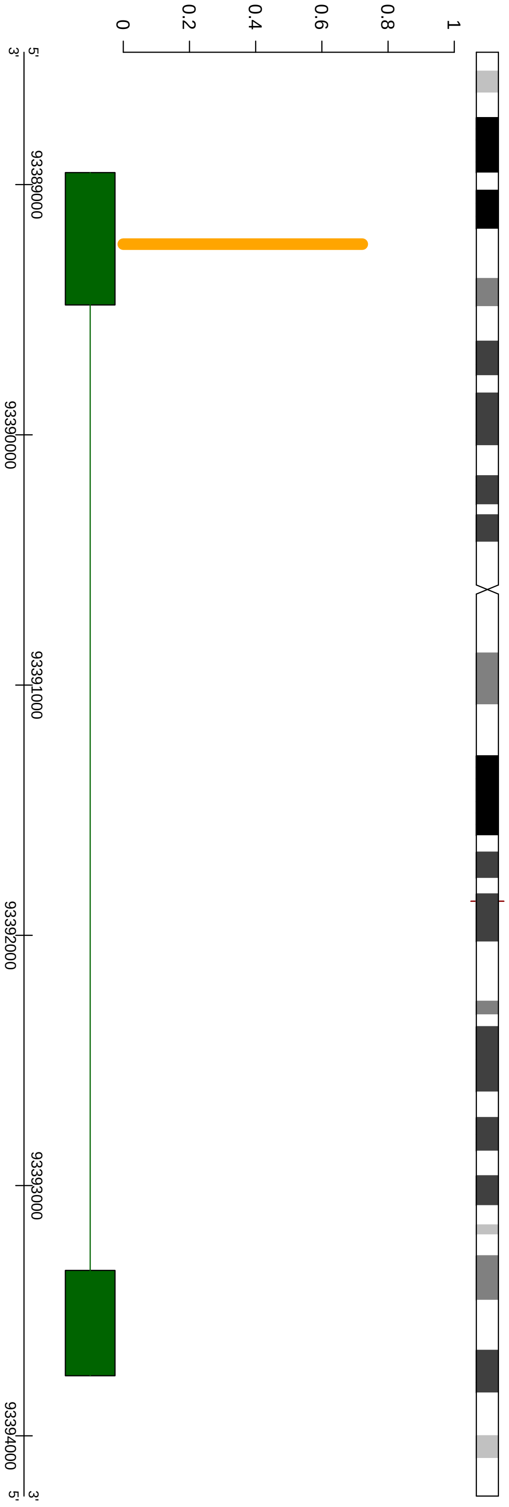

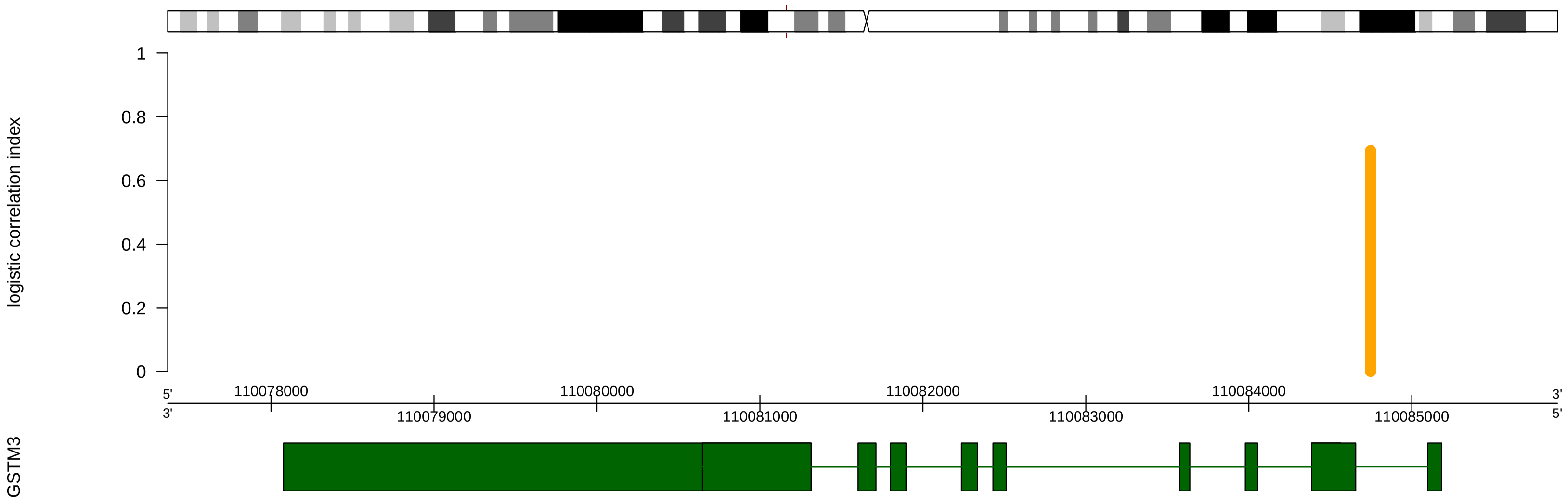
logistic correlation index
1
0.8
0.6
0.4
0.2
0
5'
3'
110078000
110079000
110080000
110081000
110082000
110083000
110084000
110085000
3'
5'
GSTM3

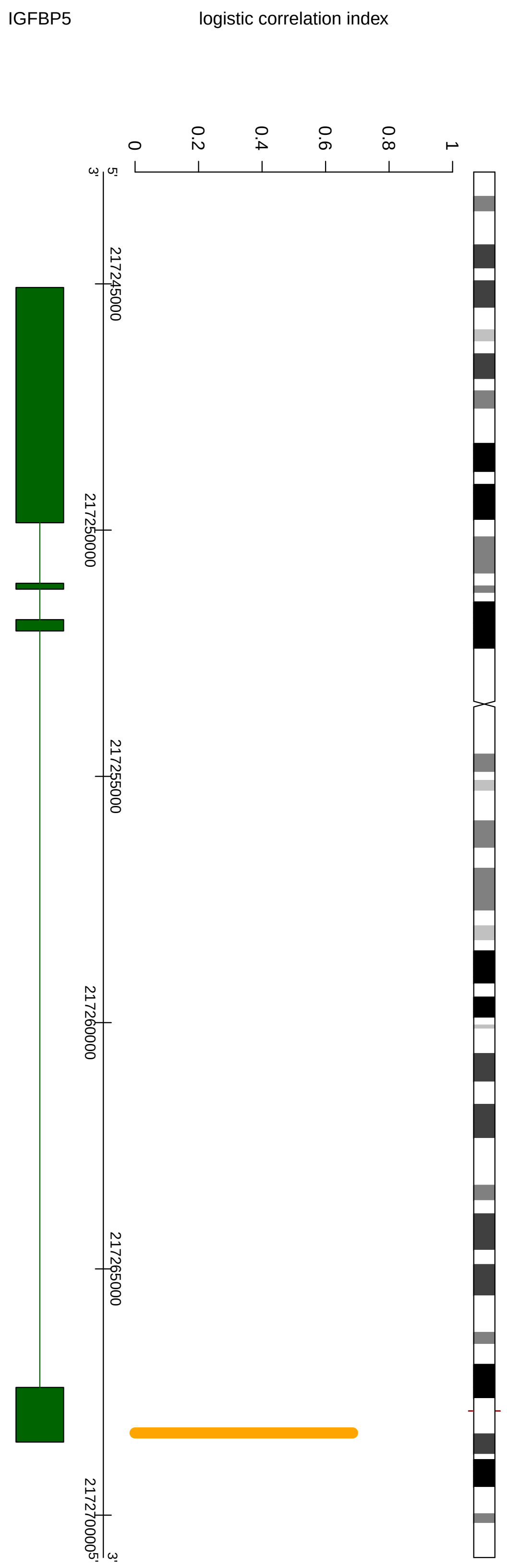
IGFBP5
logistic correlation index
0
0.2
0.4
0.6
0.8
1
5'
3'
217245000
217250000
217255000
217260000
217265000
217270000

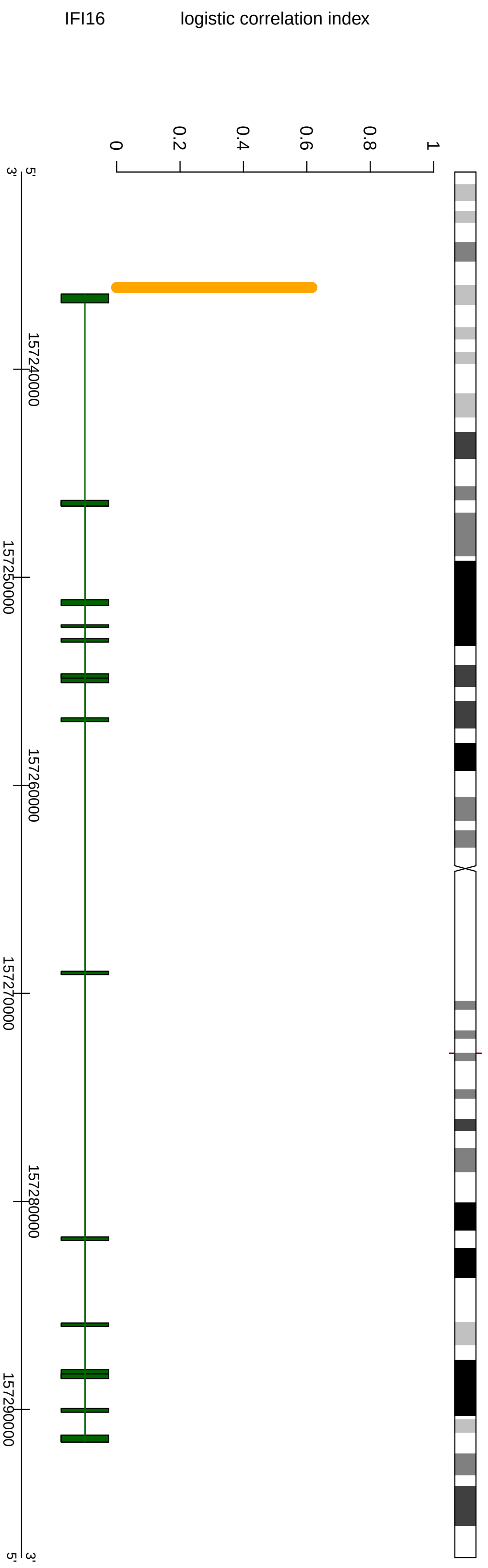
IFI16
logistic correlation index
0
0.2
0.4
0.6
0.8
1
5'
3'
157240000
157250000
157260000
157270000
157280000
157290000
3'
5'

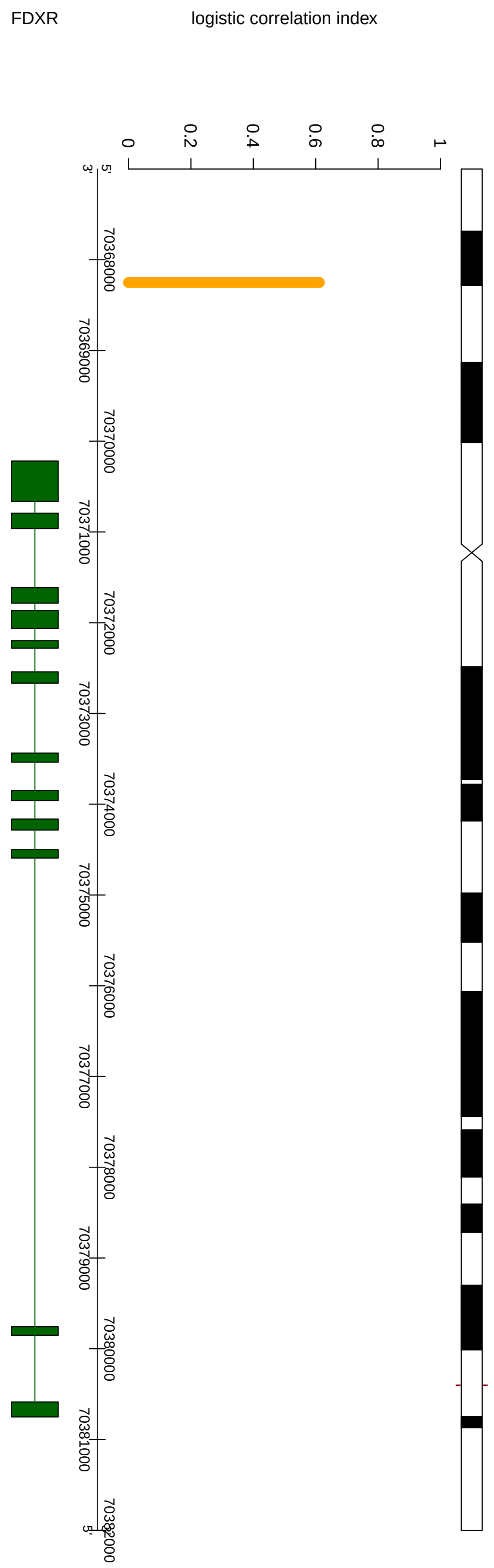
FDXR
logistic correlation index
0
0.2
0.4
0.6
0.8
1
5'
3'
70368000
70369000
70370000
70371000
70372000
70373000
70374000
70375000
70376000
70377000
70378000
70379000
70380000
70381000
70382000

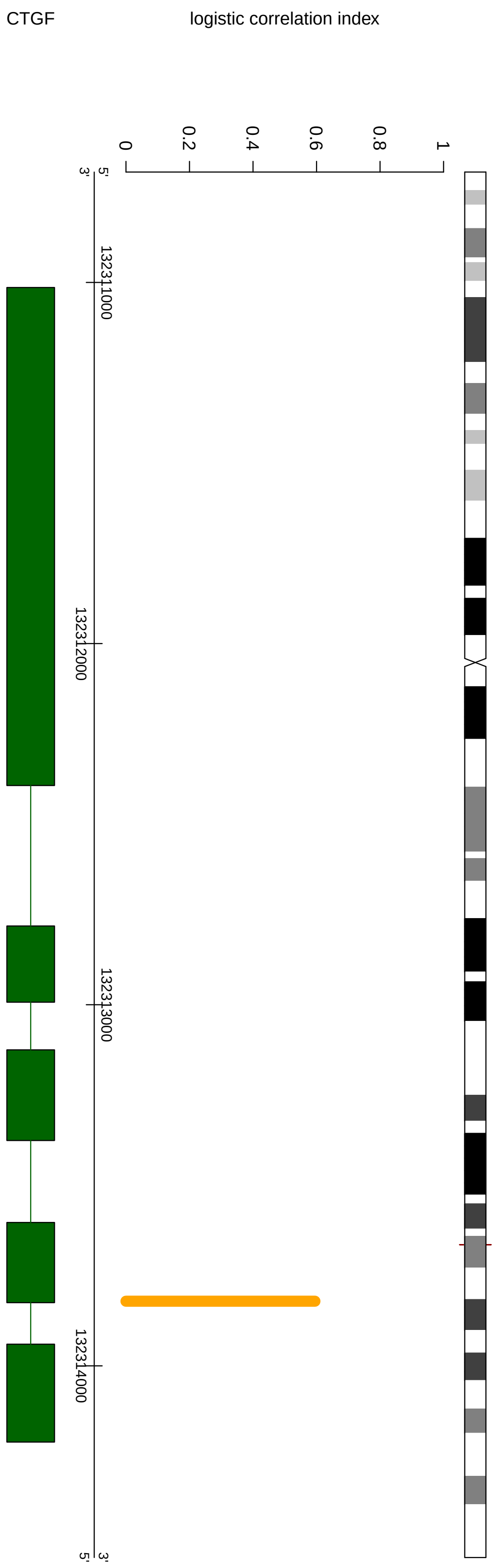
CTGF
logistic correlation index
0
0.2
0.4
0.6
0.8
1
132311000
132312000
132313000
132314000
5'
3'

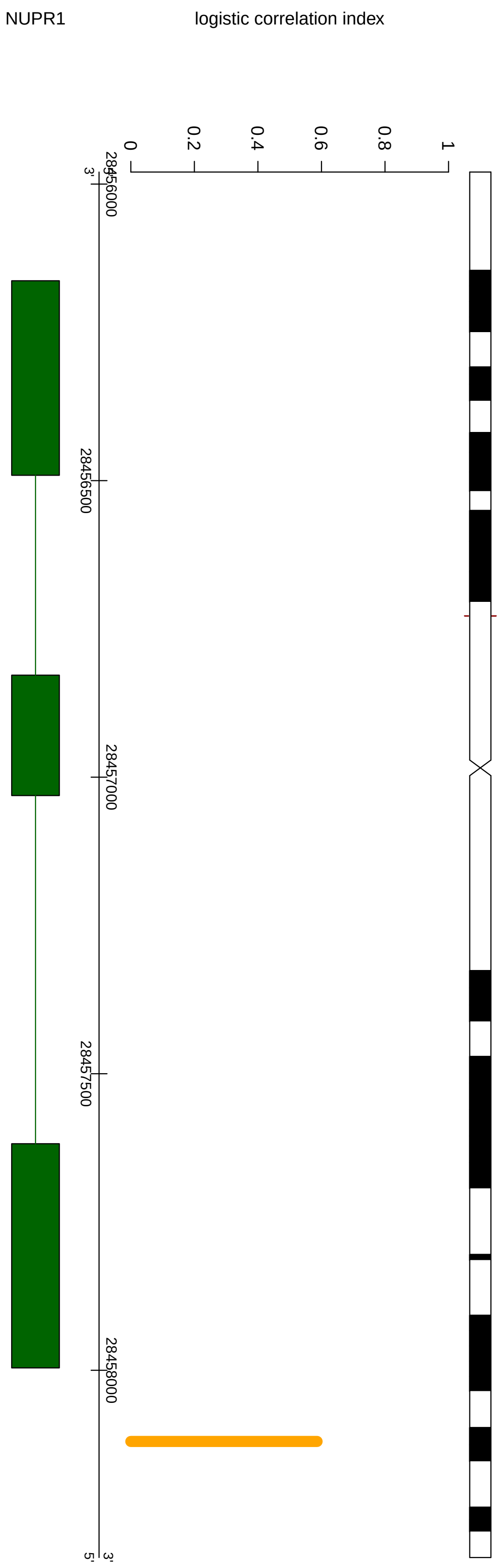
NUPR1
logistic correlation index
0
0.2
0.4
0.6
0.8
1
28456000
28456500
28457000
28457500
28458000
3'
5'
3'

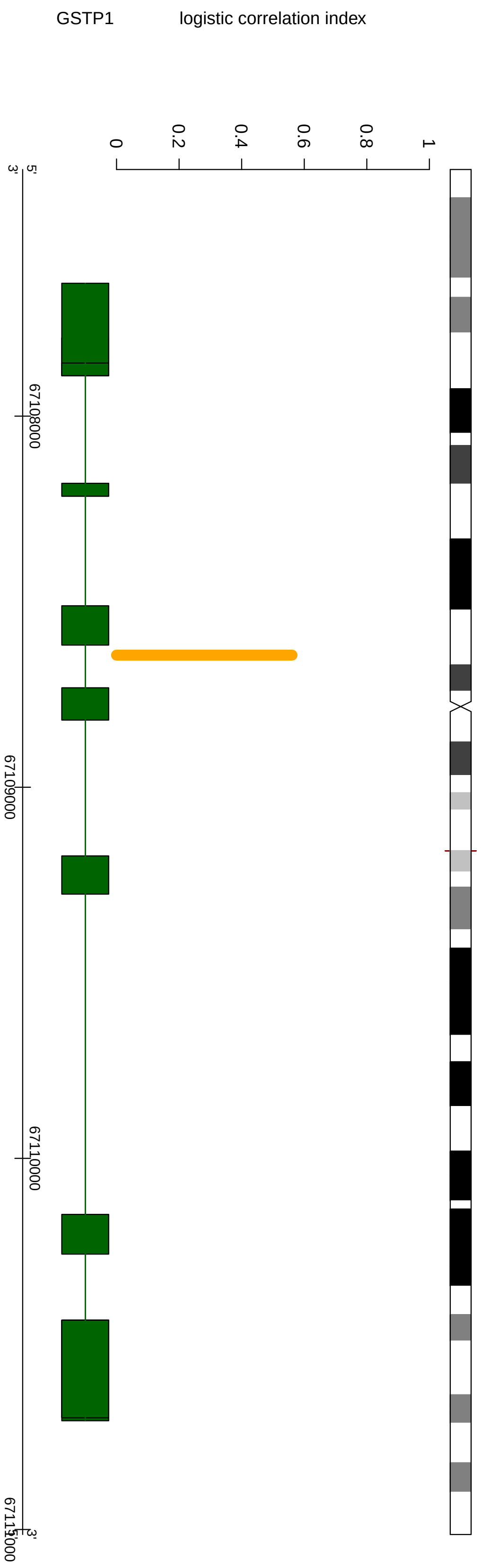
GSTP1
logistic correlation index
0
0.2
0.4
0.6
0.8
1
5'
3'
67108000
67109000
67110000
67111000
3'

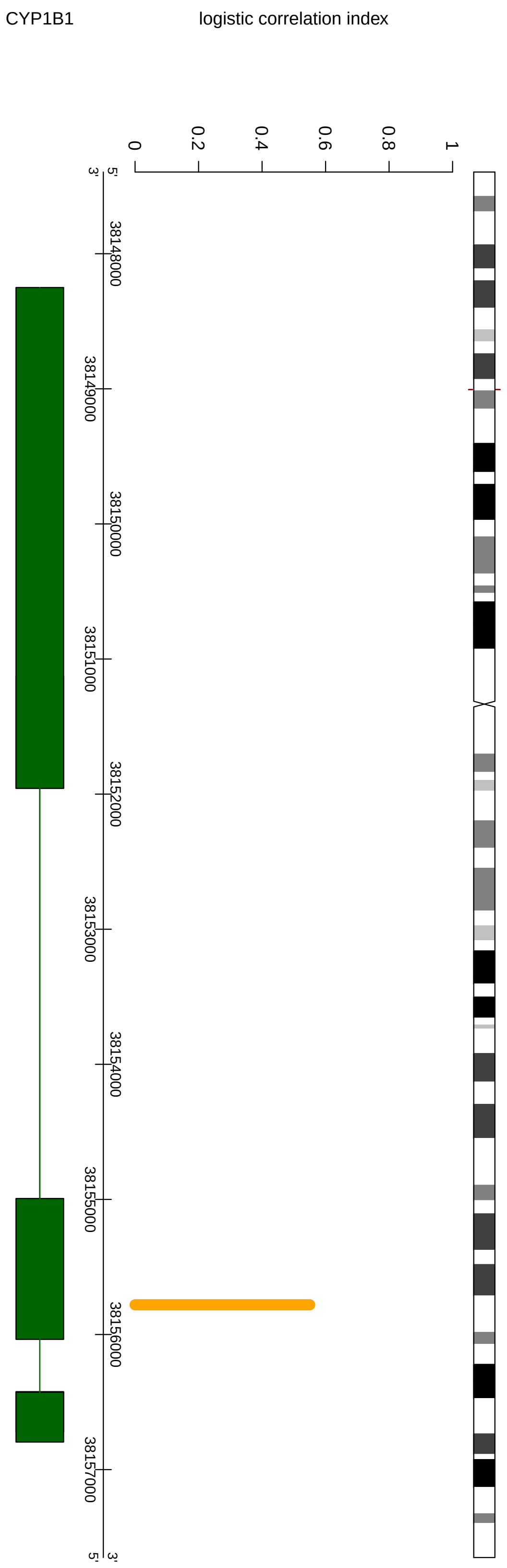
CYP1B1
logistic correlation index
0
0.2
0.4
0.6
0.8
1
5'
3'
38148000
38149000
38150000
38151000
38152000
38153000
38154000
38155000
38156000
38157000

TOP2A

logistic correlation index

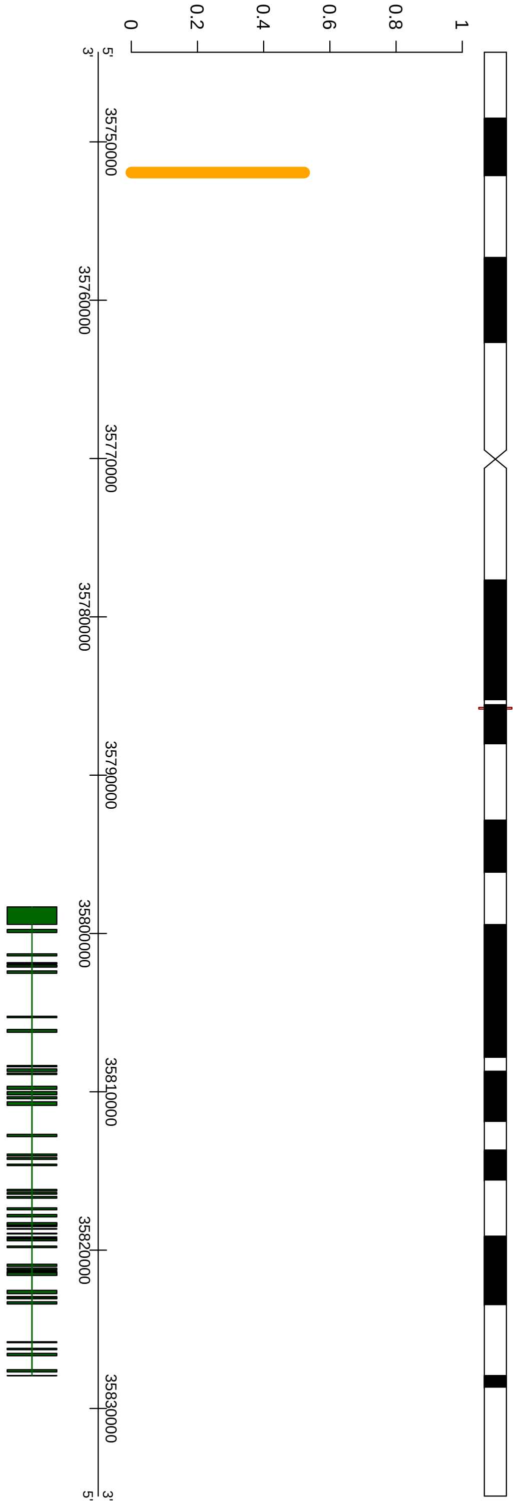

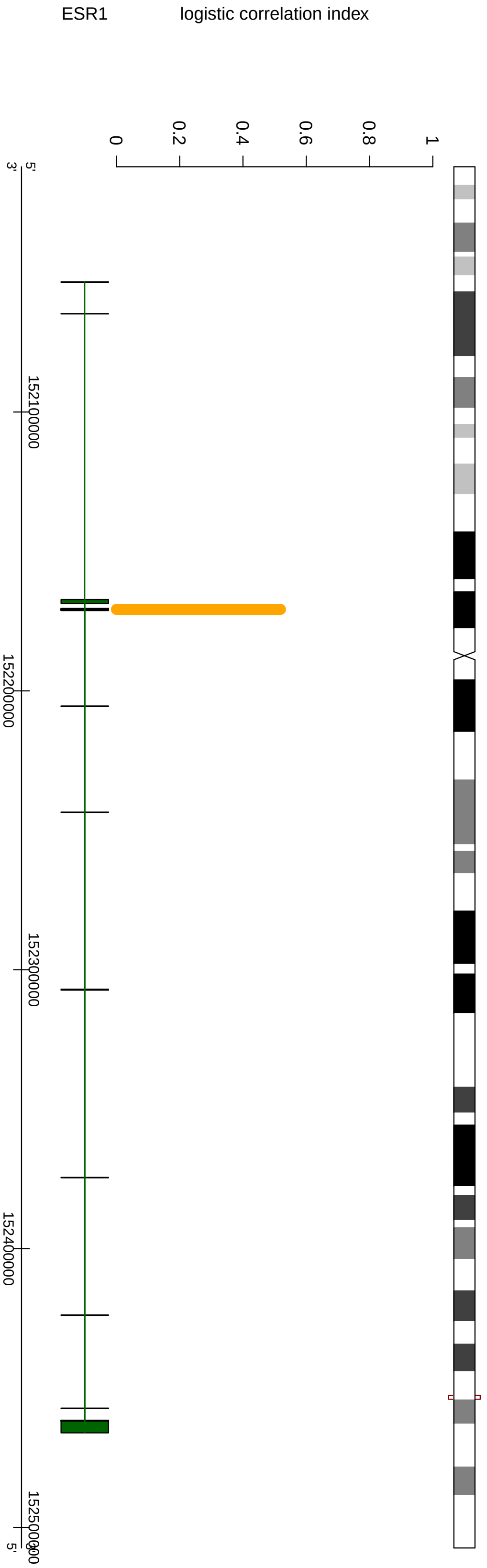
ESR1
logistic correlation index
0
0.2
0.4
0.6
0.8
1
5'
3'
152100000
152200000
152300000
152400000
152500000
5'

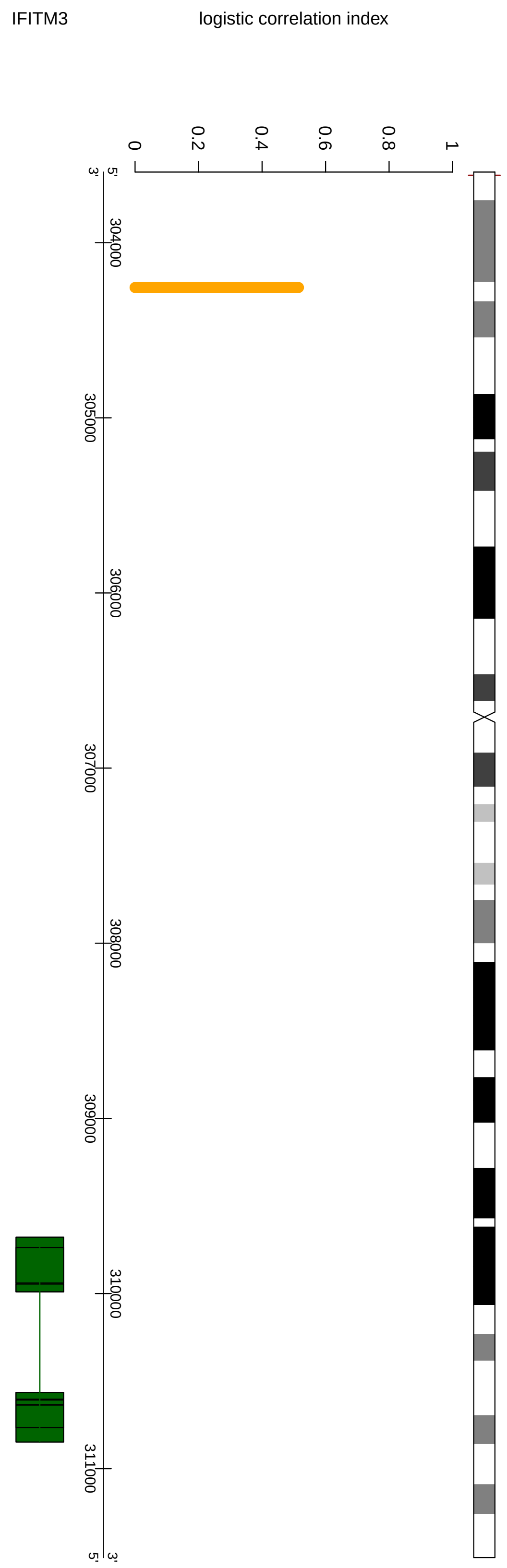
IFITM3
logistic correlation index
0
0.2
0.4
0.6
0.8
1
5'
3'
304000
305000
306000
307000
308000
309000
310000
311000

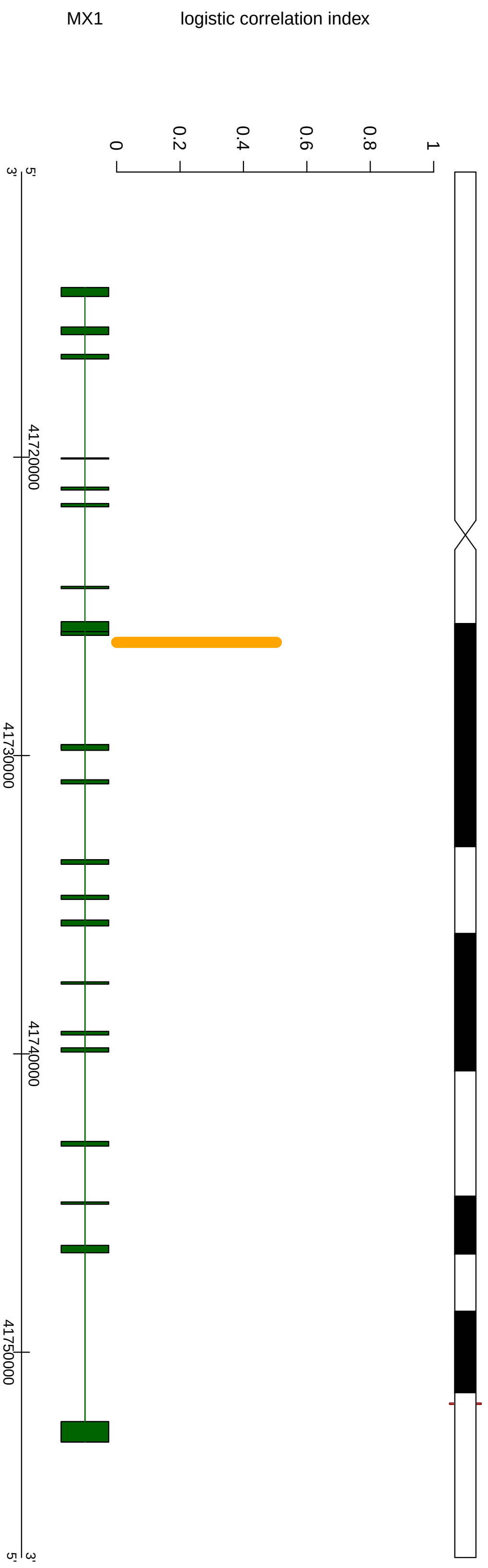

MX1
logistic correlation index
0
0.2
0.4
0.6
0.8
1
41720000
41730000
41740000
41750000
5'
3'

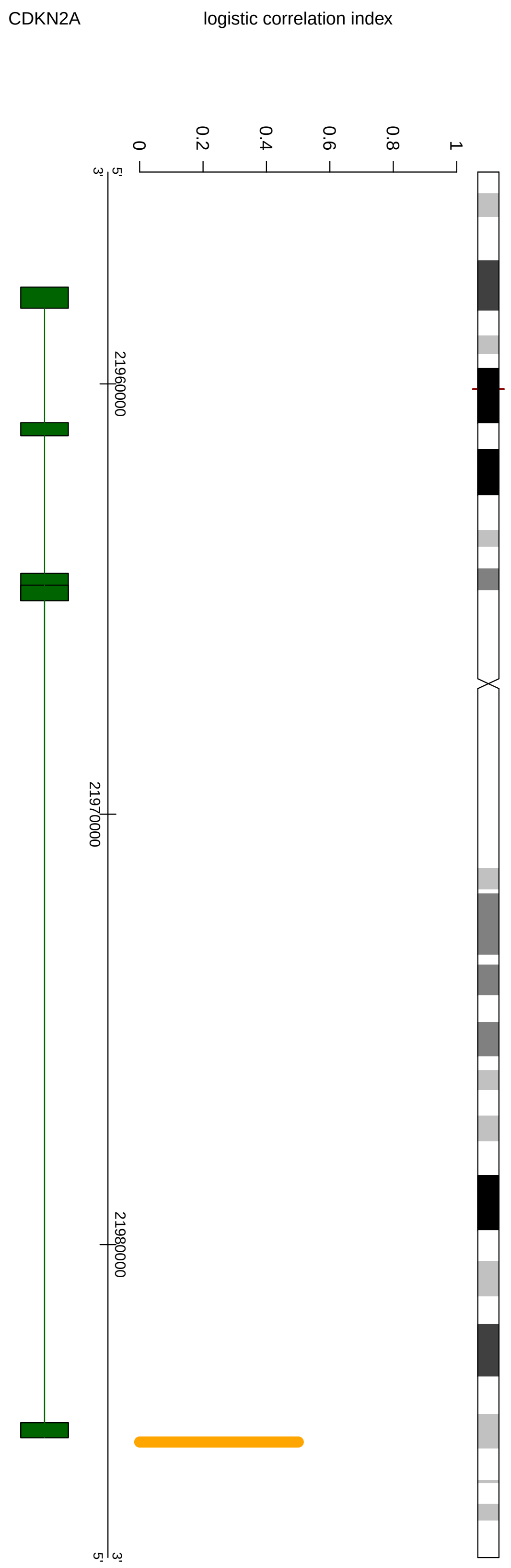
CDKN2A
logistic correlation index
0
0.2
0.4
0.6
0.8
1
5'
3'
21960000
21970000
21980000
3'
5'

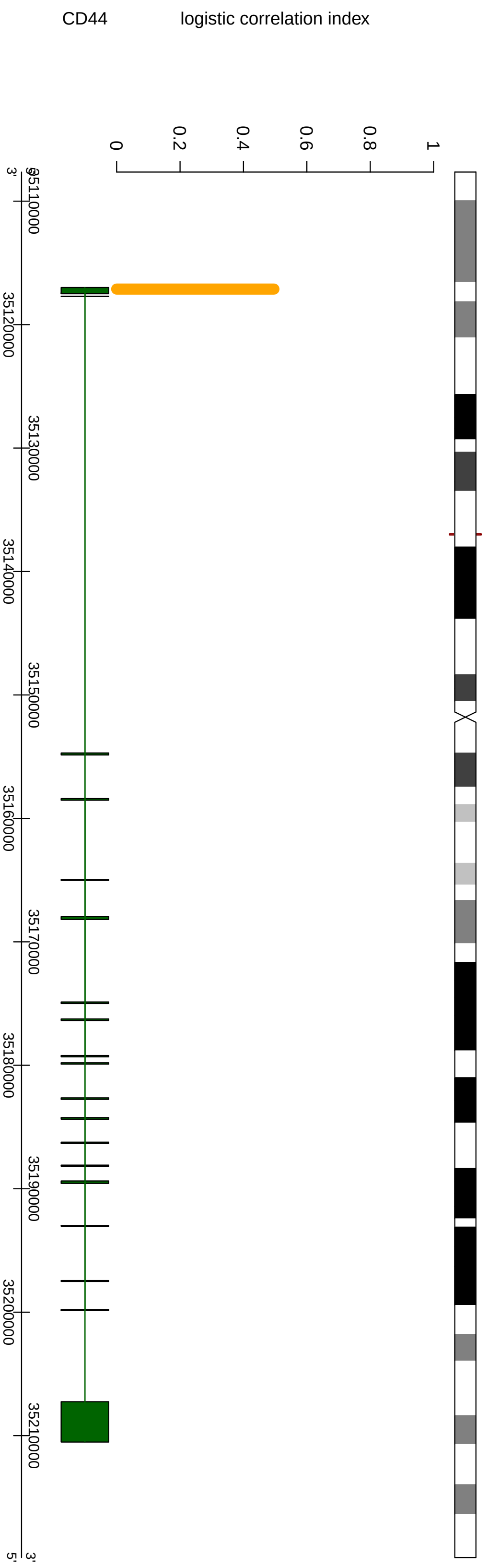
CD44
logistic correlation index
0
0.2
0.4
0.6
0.8
1
35110000
35120000
35130000
35140000
35150000
35160000
35170000
35180000
35190000
35200000
35210000
3'
5'

MTHFD1 logistic correlation index

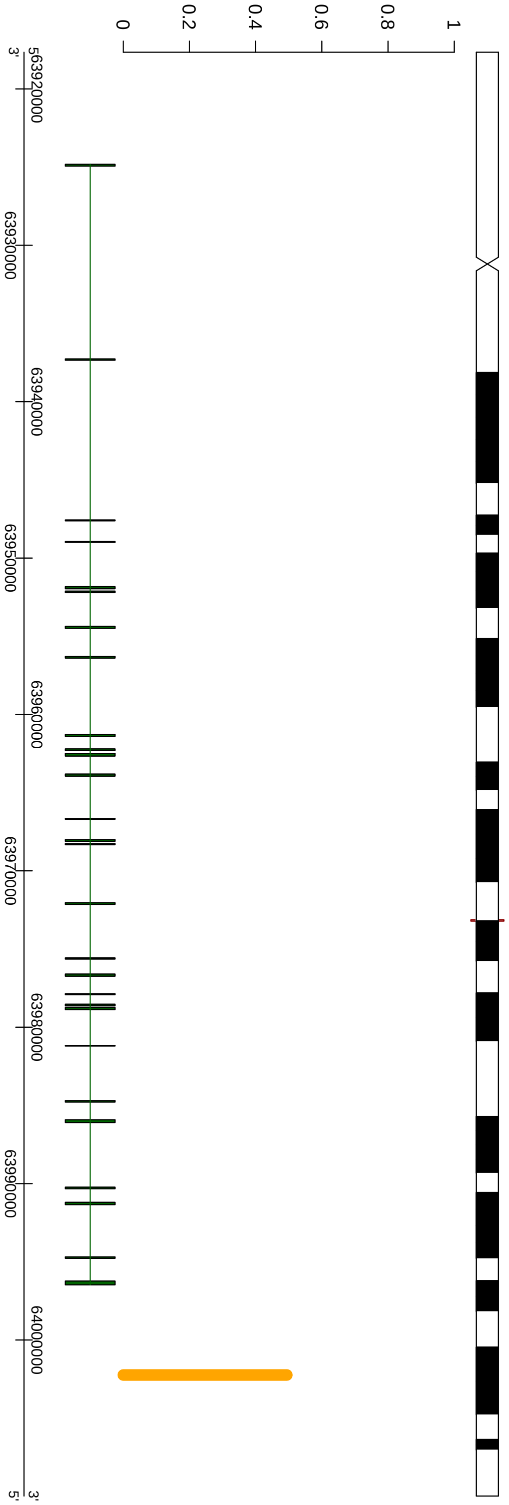

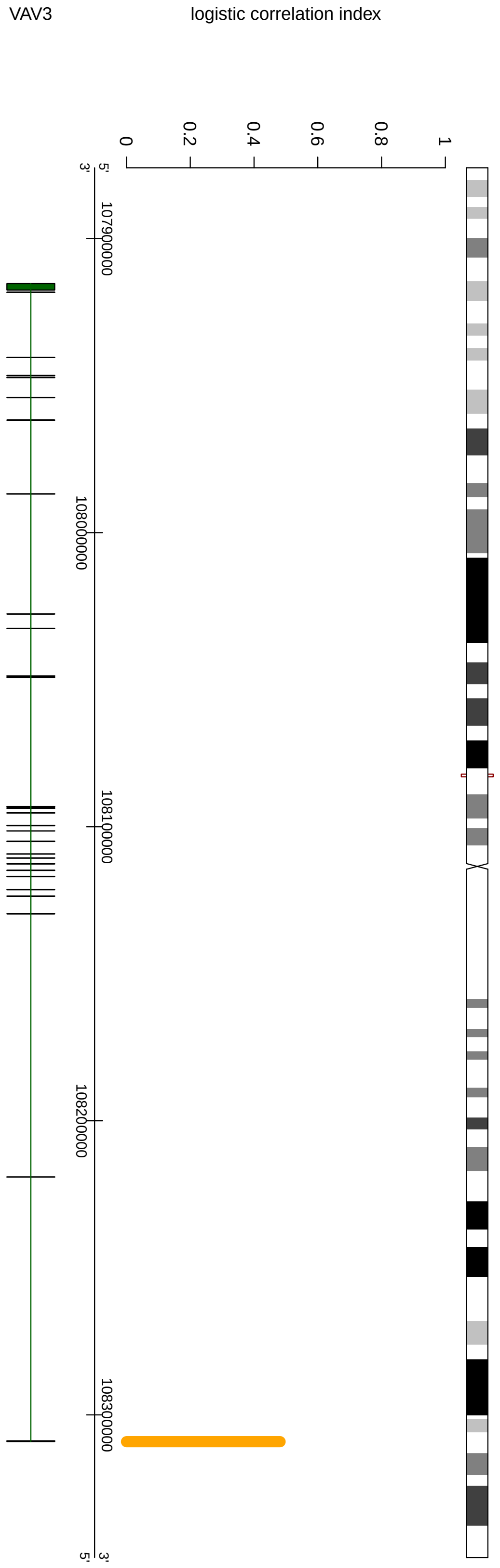
VAV3
logistic correlation index
0
0.2
0.4
0.6
0.8
1
5'
3'
107900000
108000000
108100000
108200000
108300000
3'
5'

TFAP2A logistic correlation index

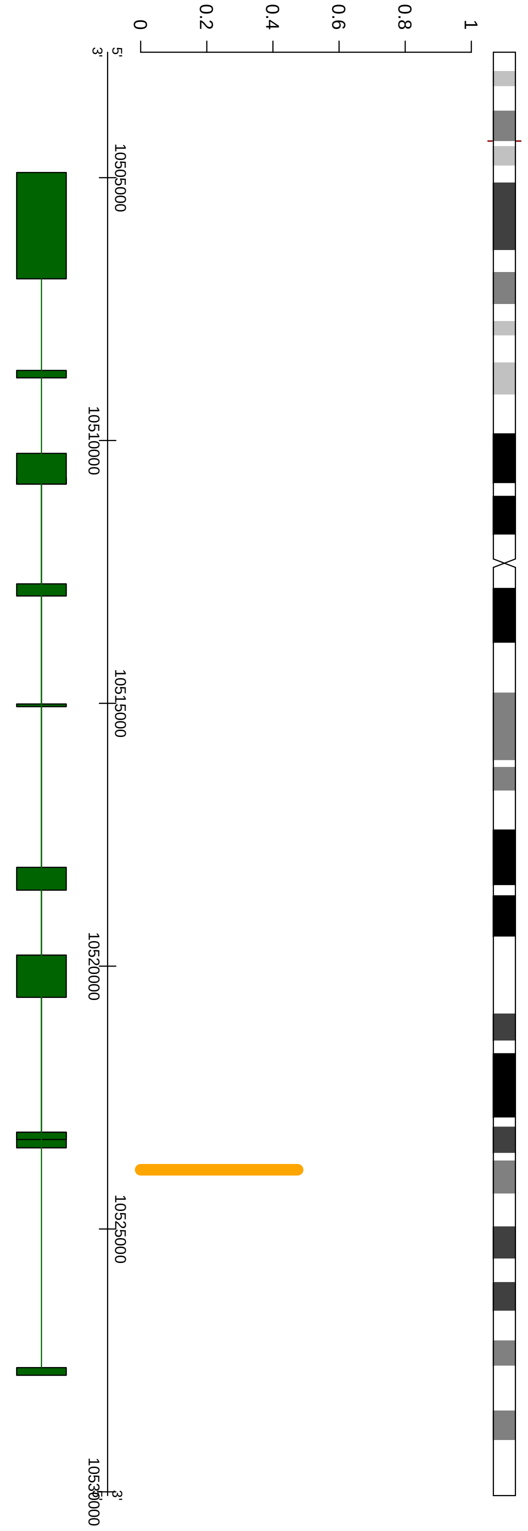

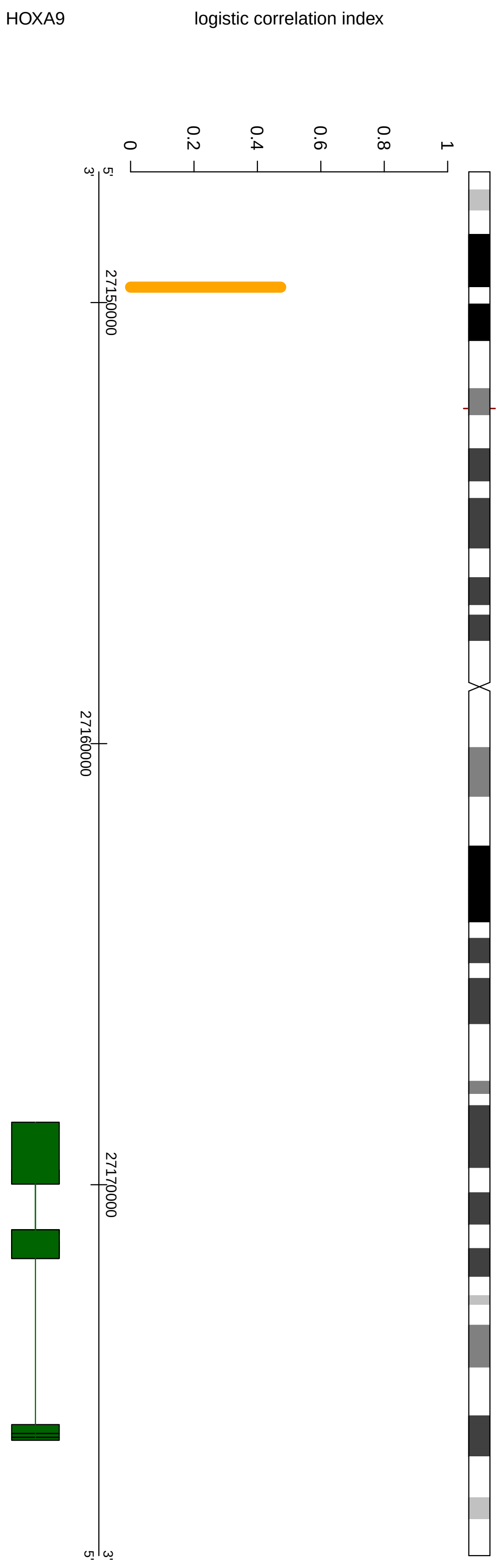
HOXA9
logistic correlation index
0
0.2
0.4
0.6
0.8
1
5'
3'
27150000
27160000
27170000
3'
5'

DHRS2 logistic correlation index

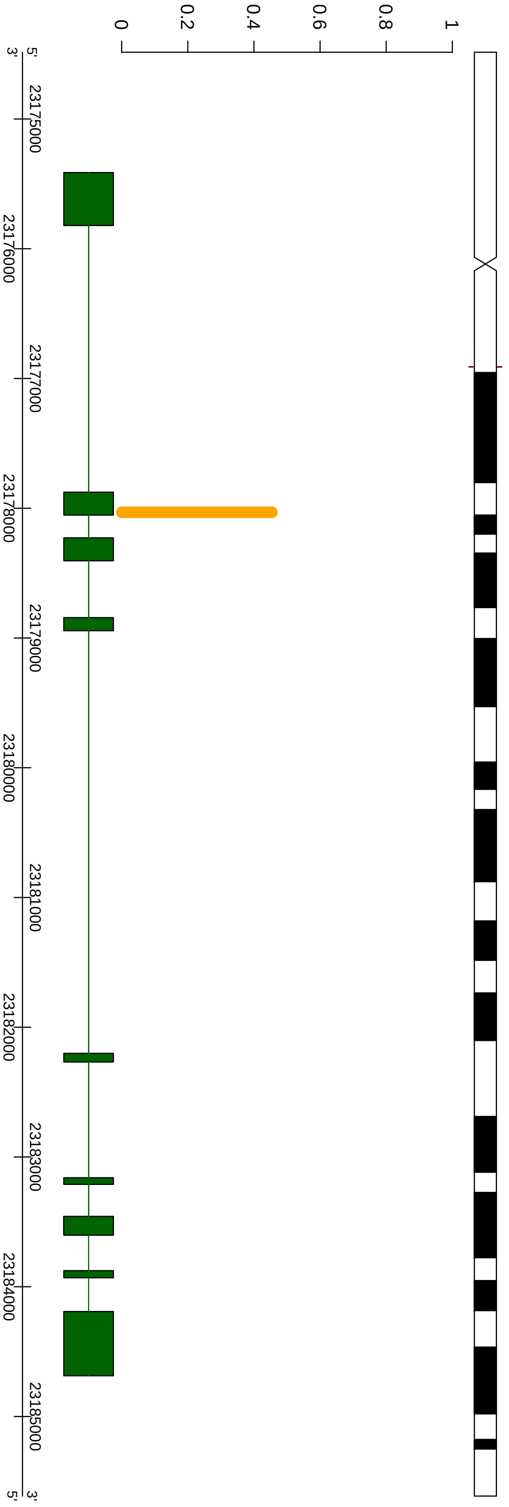

CBFA2T3 logistic correlation index

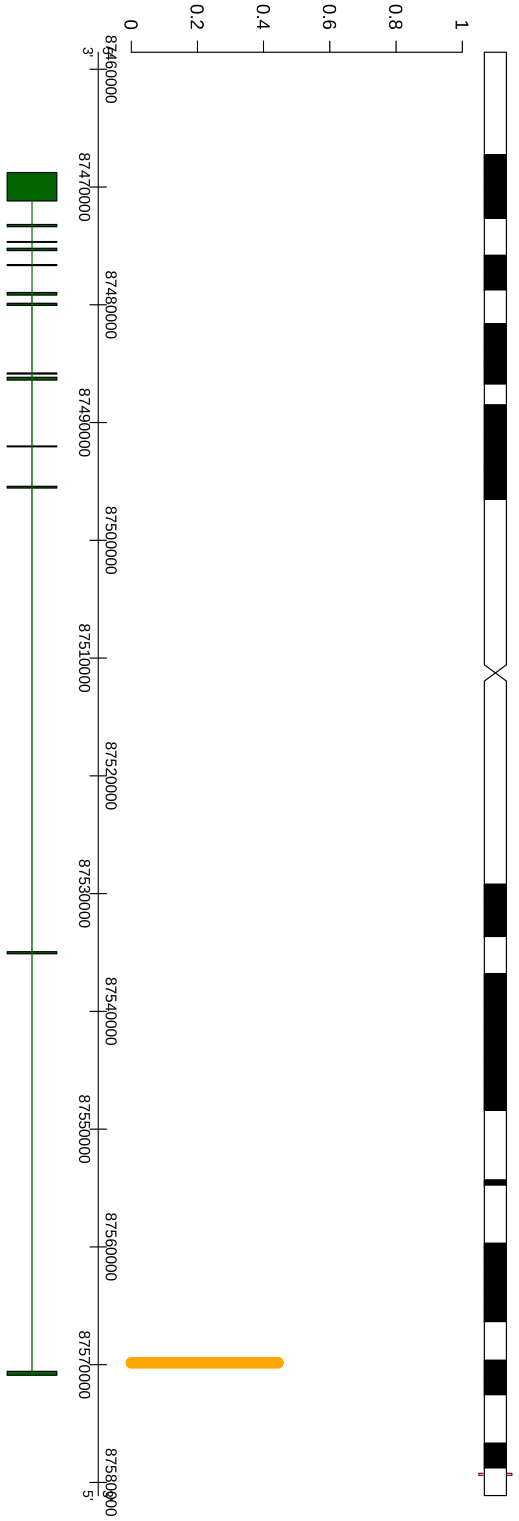

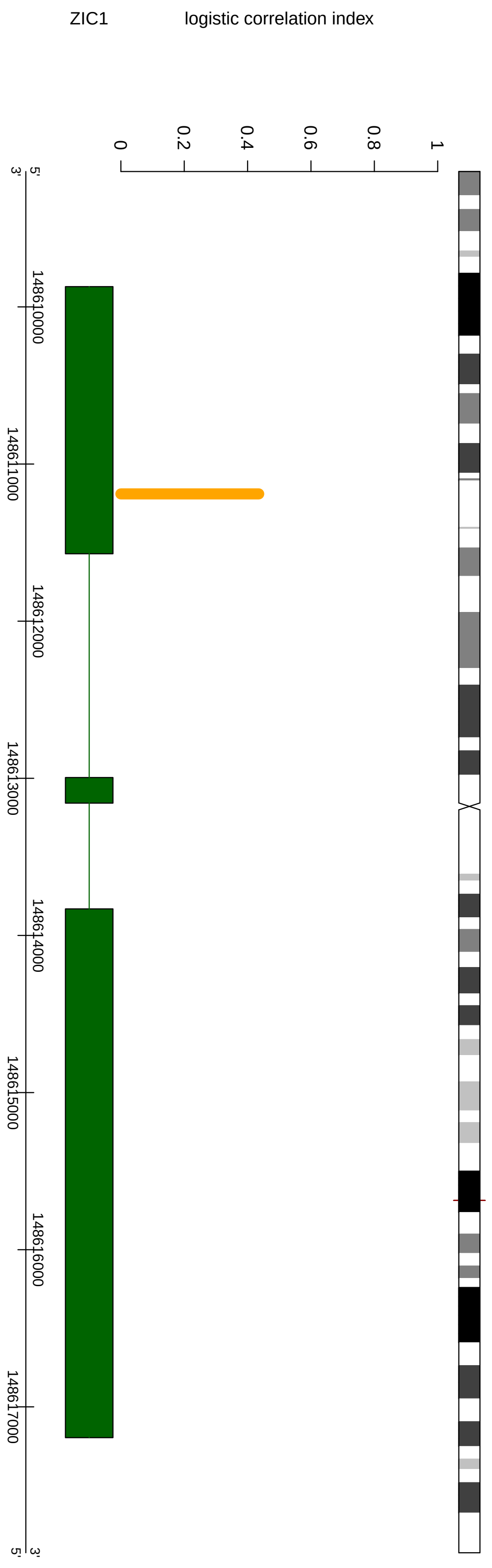
ZIC1
logistic correlation index
0
0.2
0.4
0.6
0.8
1
5'
3'
148610000
148611000
148612000
148613000
148614000
148615000
148616000
148617000
3'
5'

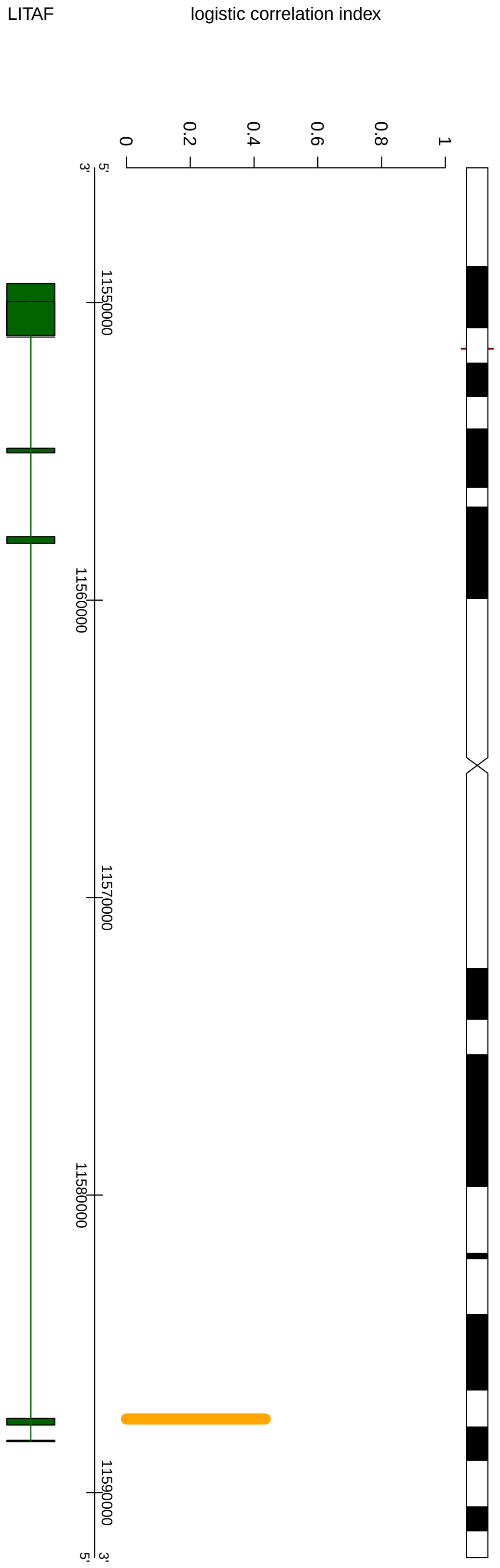
LITAF
logistic correlation index
0
0.2
0.4
0.6
0.8
1
5'
3'
11550000
11560000
11570000
11580000
11590000
3'
5'

ADAM12 logistic correlation index

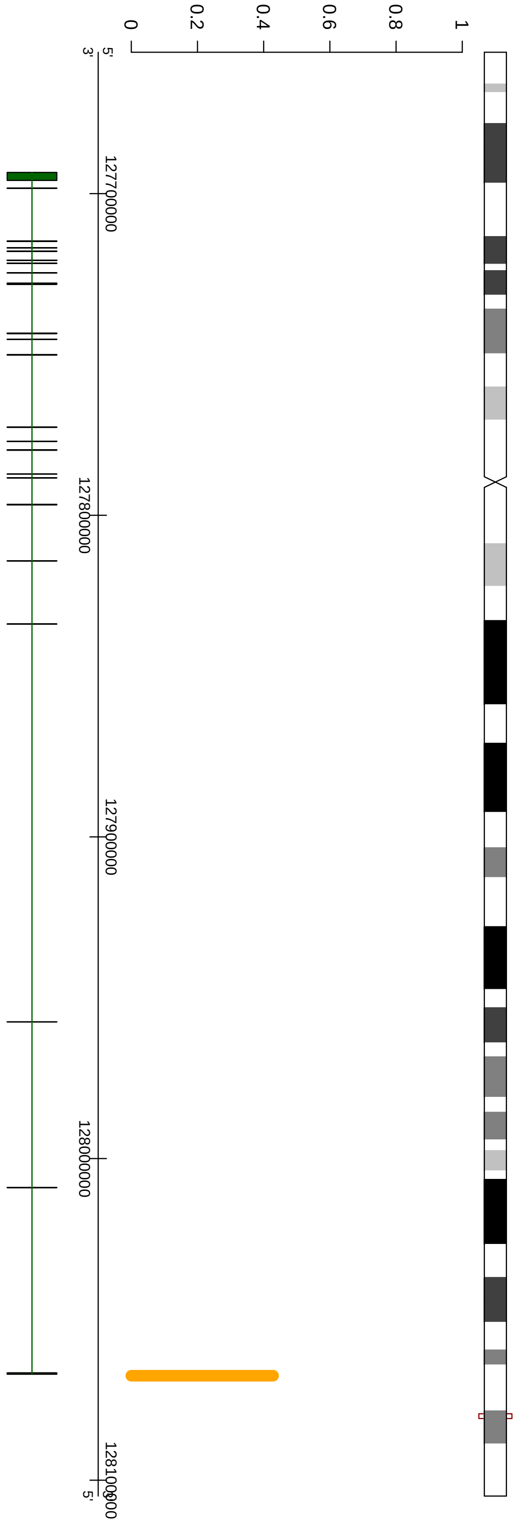

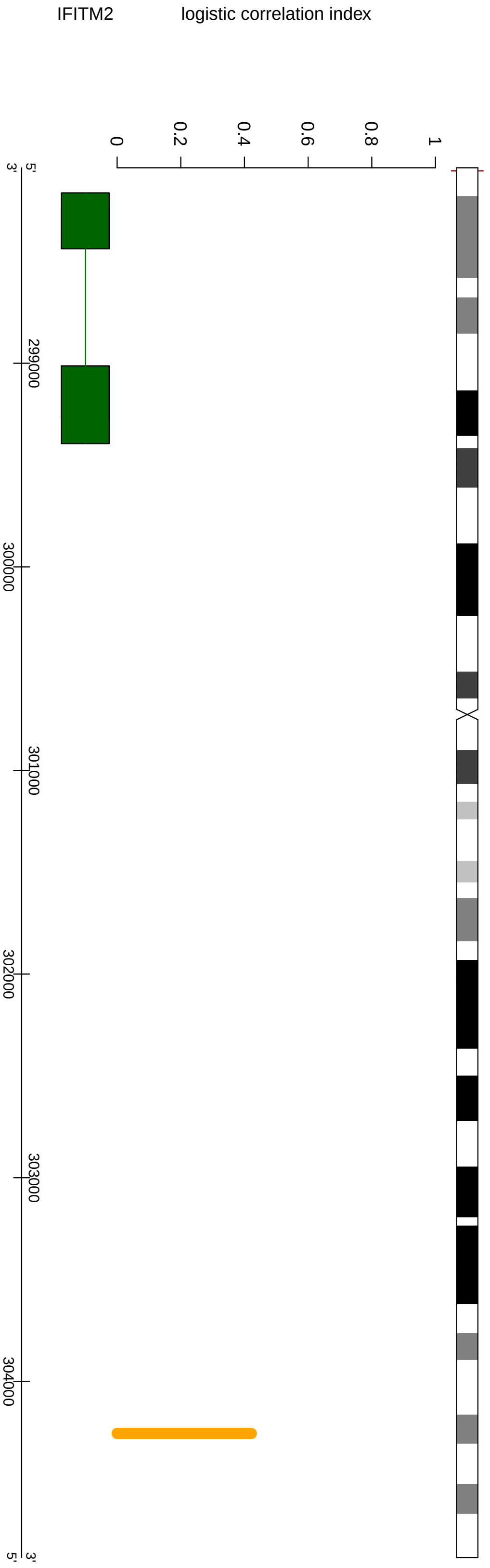
IFITM2
logistic correlation index
0
0.2
0.4
0.6
0.8
1
5'
3'
299000
300000
301000
302000
303000
304000
3'
5'

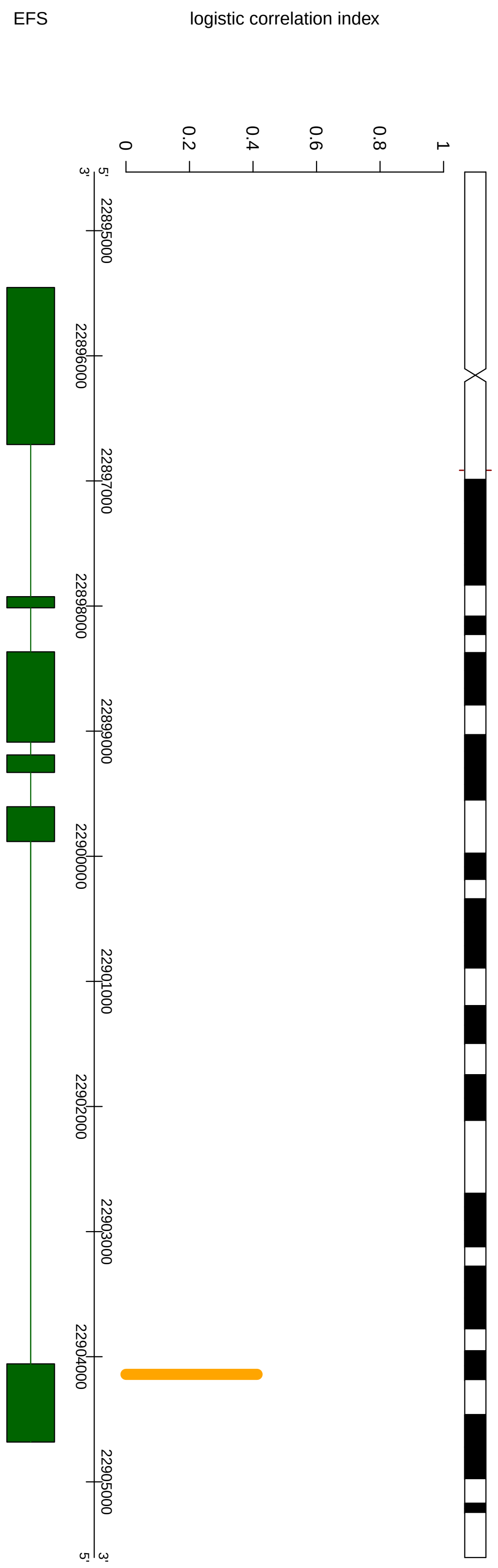
EFS
logistic correlation index
0
0.2
0.4
0.6
0.8
1
5'
3'
22895000
22896000
22897000
22898000
22899000
22900000
22901000
22902000
22903000
22904000
22905000
3'
5'

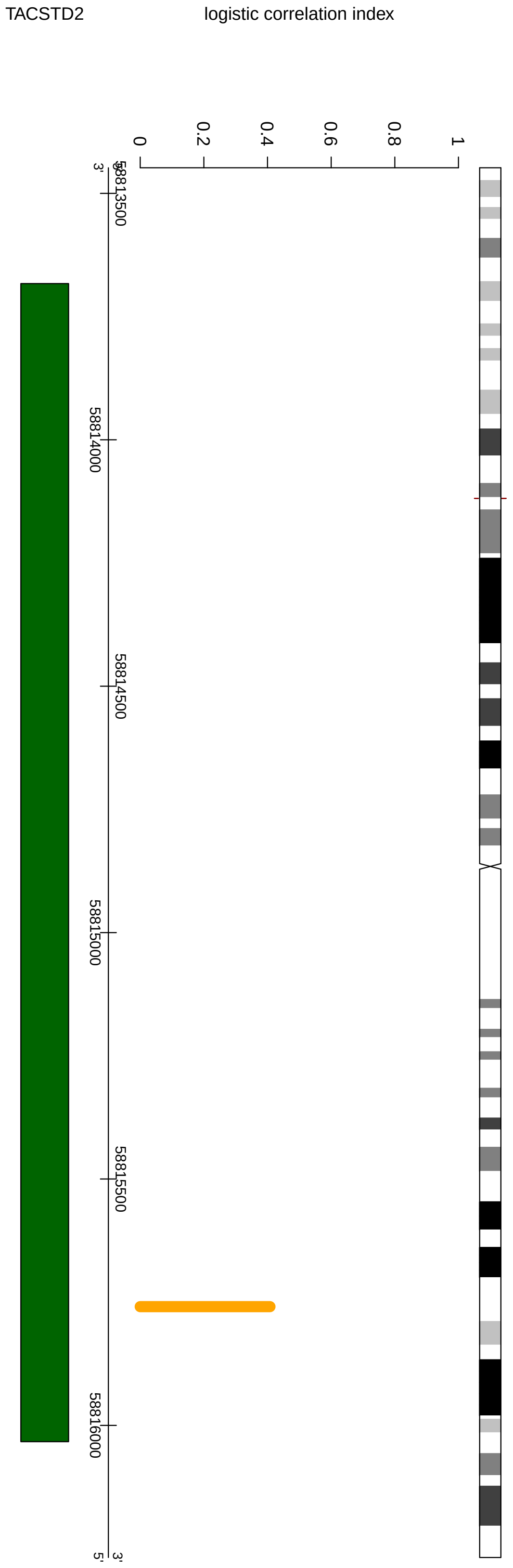
TACSTD2
logistic correlation index
0
0.2
0.4
0.6
0.8
1
58813500
58814000
58814500
58815000
58815500
58816000
3'
5'

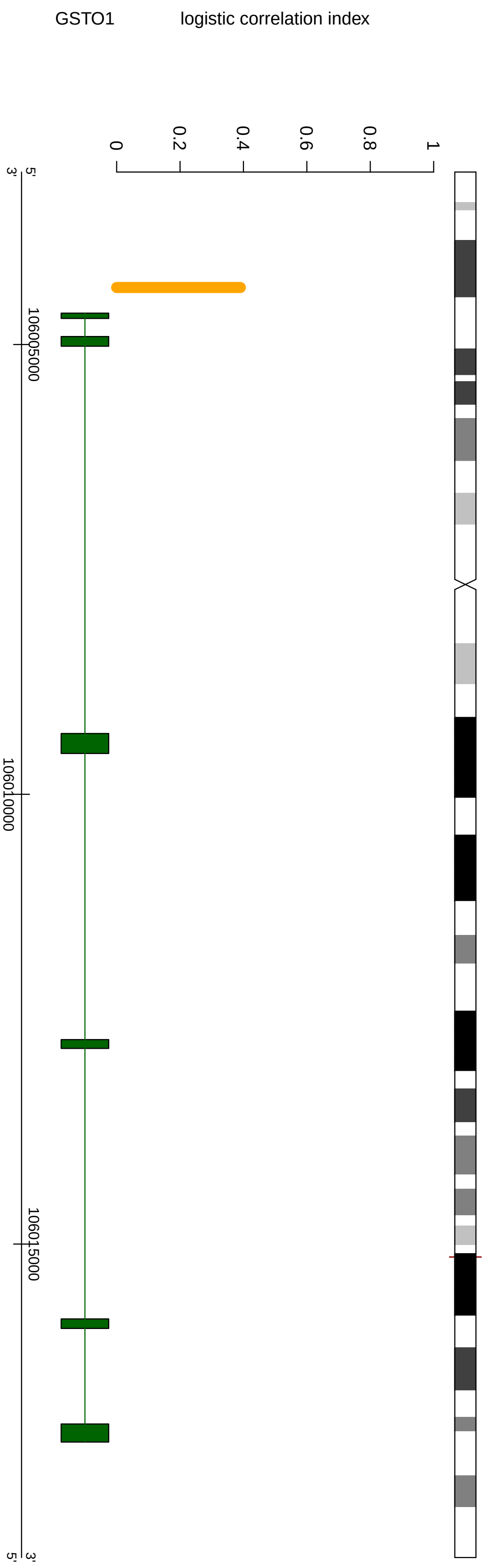
GSTO1
logistic correlation index
0
0.2
0.4
0.6
0.8
1
5'
3'
106005000
106010000
106015000
3'
5'

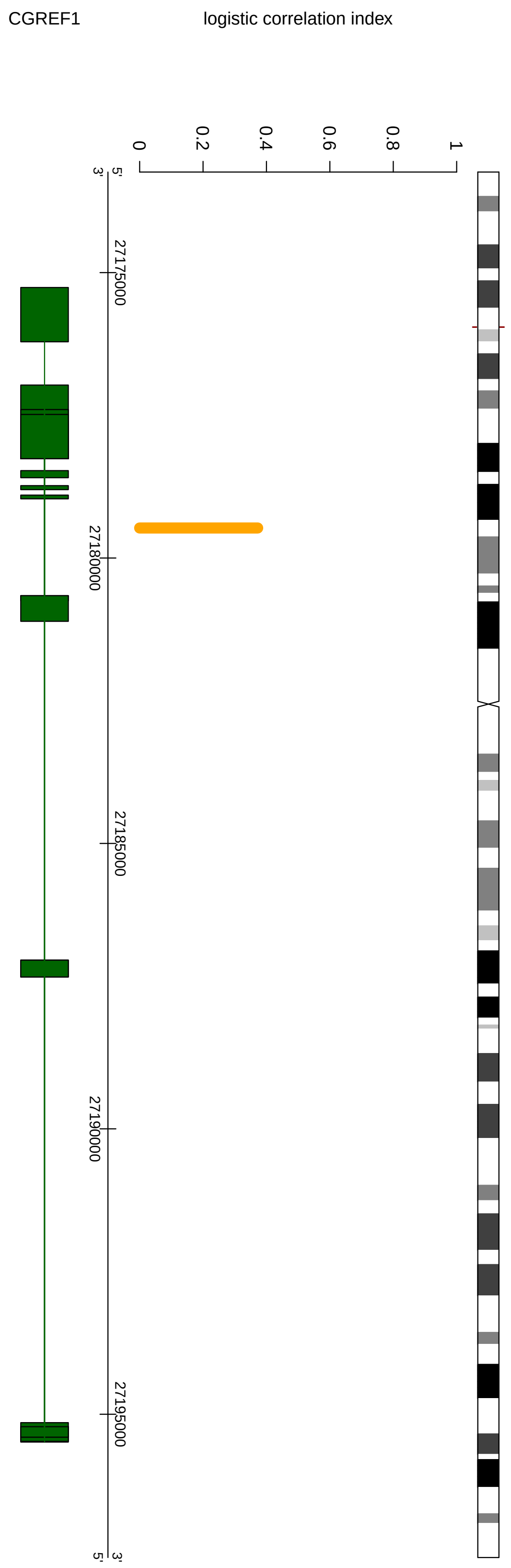
CGREF1
logistic correlation index
0
0.2
0.4
0.6
0.8
1
5'
3'
27175000
27180000
27185000
27190000
27195000
3'
5'

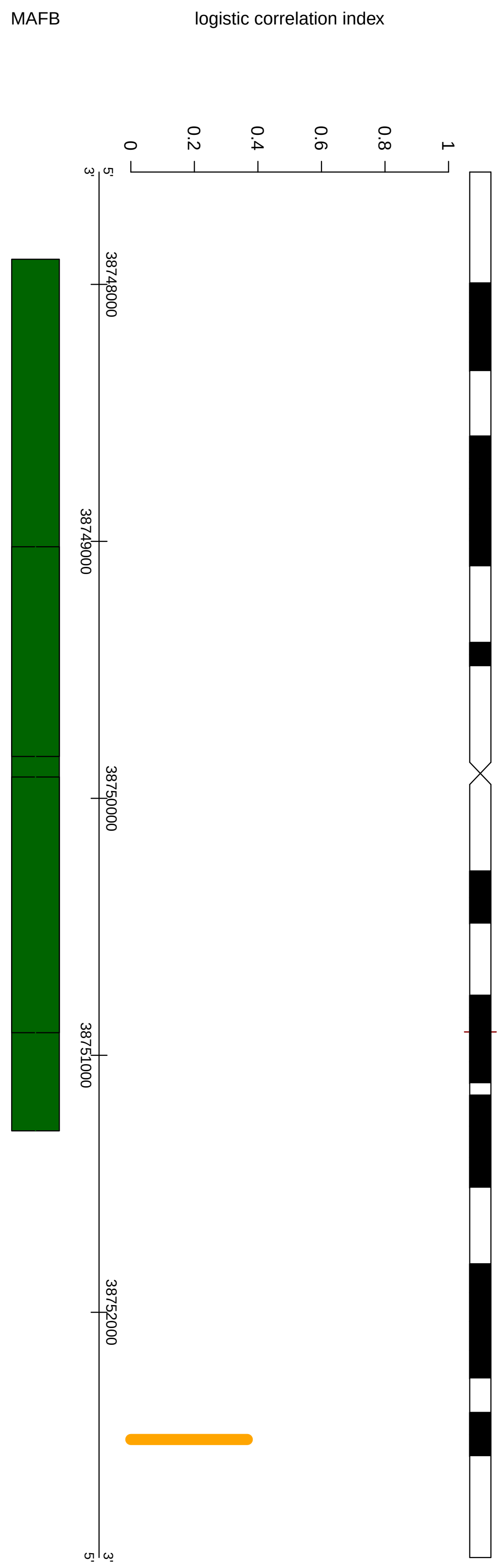
MAFB
logistic correlation index
0
0.2
0.4
0.6
0.8
1
5'
3'
38748000
38749000
38750000
38751000
38752000
3'
5'

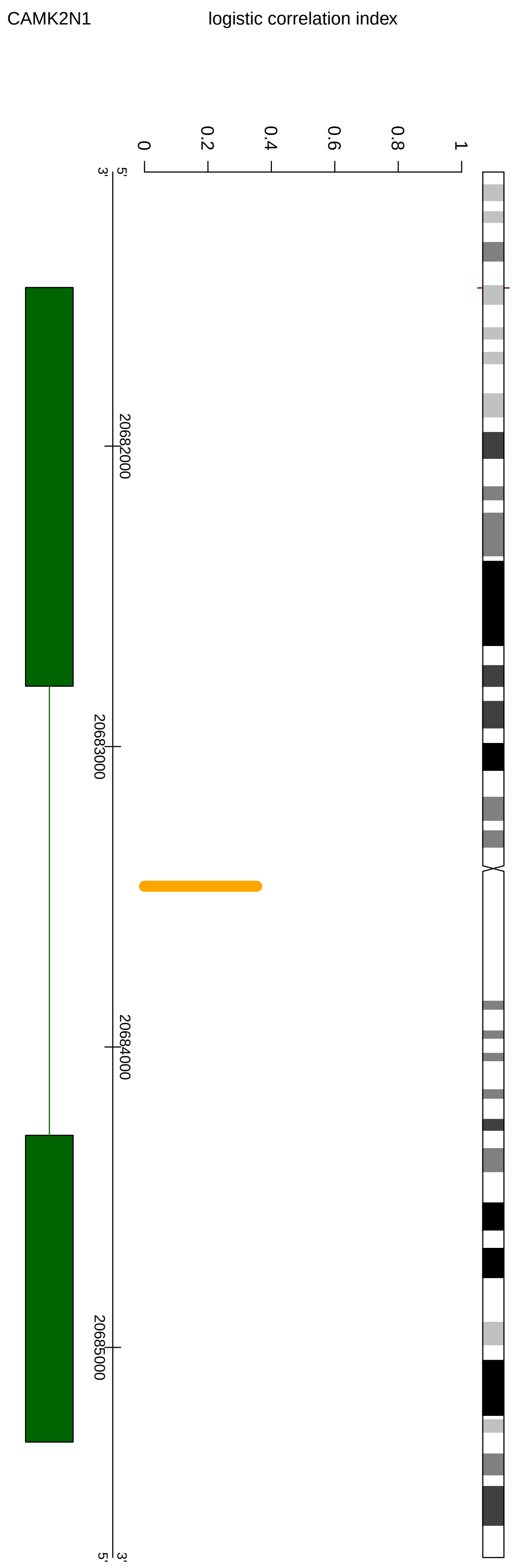
CAMK2N1
logistic correlation index
0
0.2
0.4
0.6
0.8
1
5'
3'
20682000
20683000
20684000
20685000
3'
5'

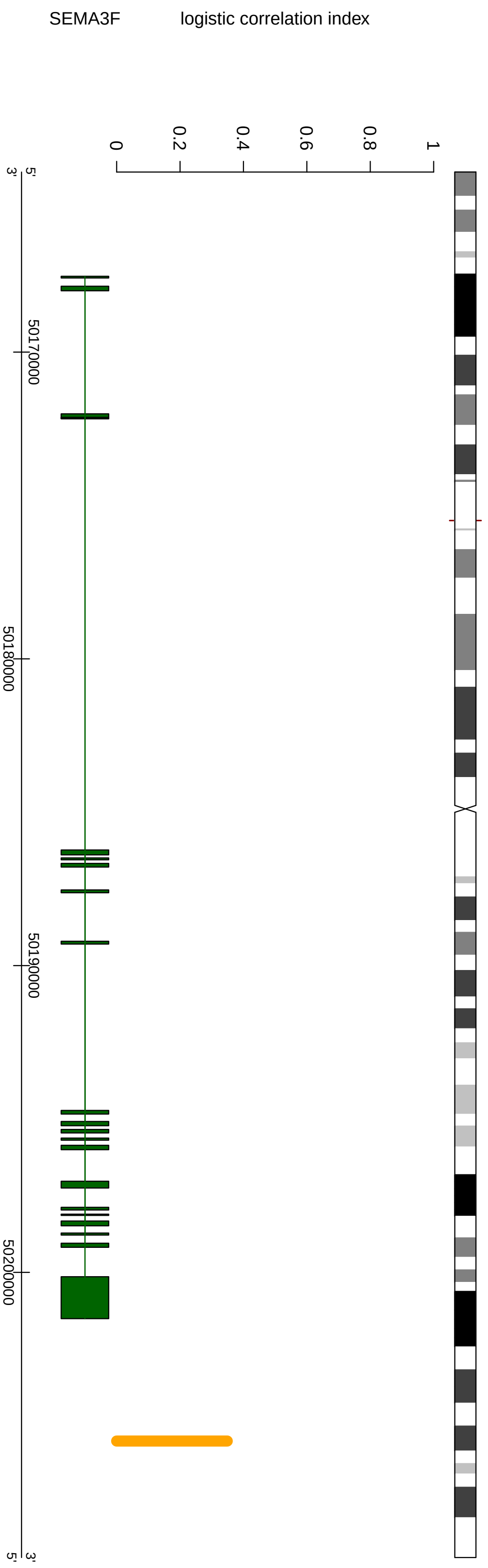
SEMA3F
logistic correlation index
0
0.2
0.4
0.6
0.8
1
5'
3'
50170000
50180000
50190000
50200000
3'
5'

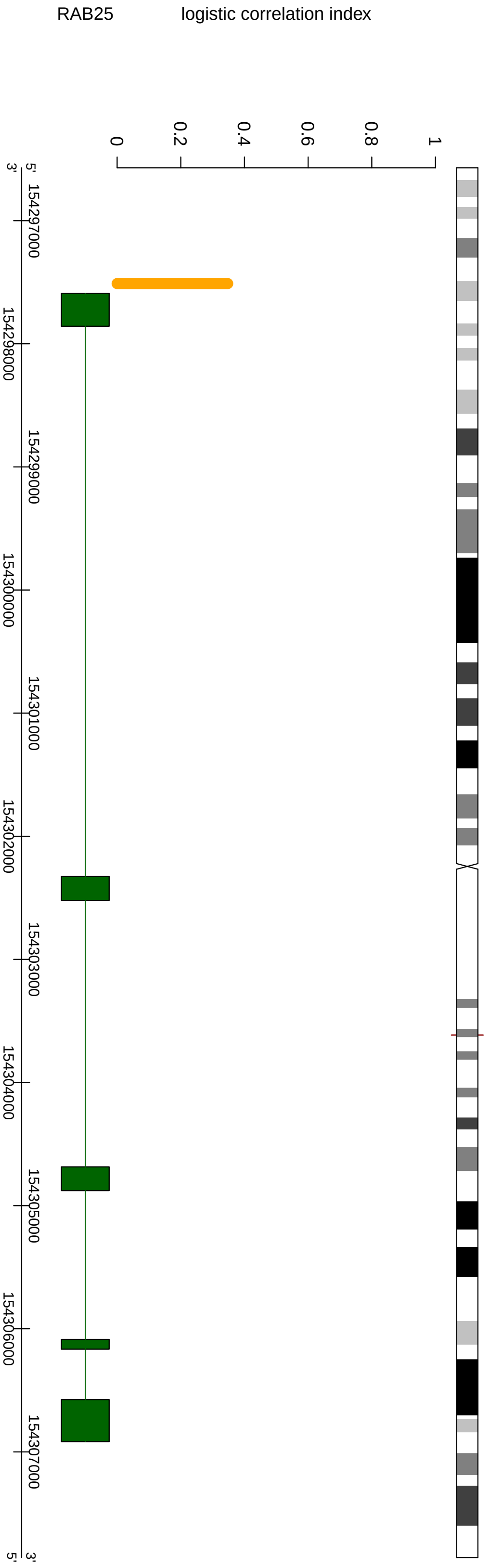
RAB25
logistic correlation index
0
0.2
0.4
0.6
0.8
1
154297000
154298000
154299000
154300000
154301000
154302000
154303000
154304000
154305000
154306000
154307000
5'
3'
3'
5'

ANXA13 logistic correlation index

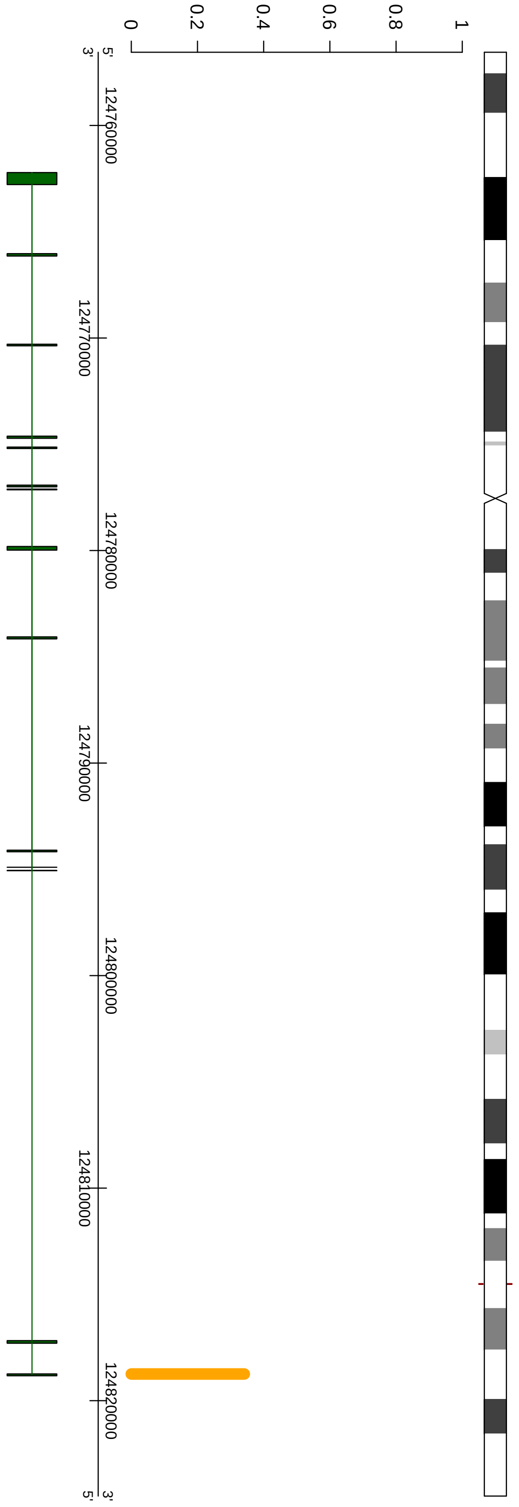

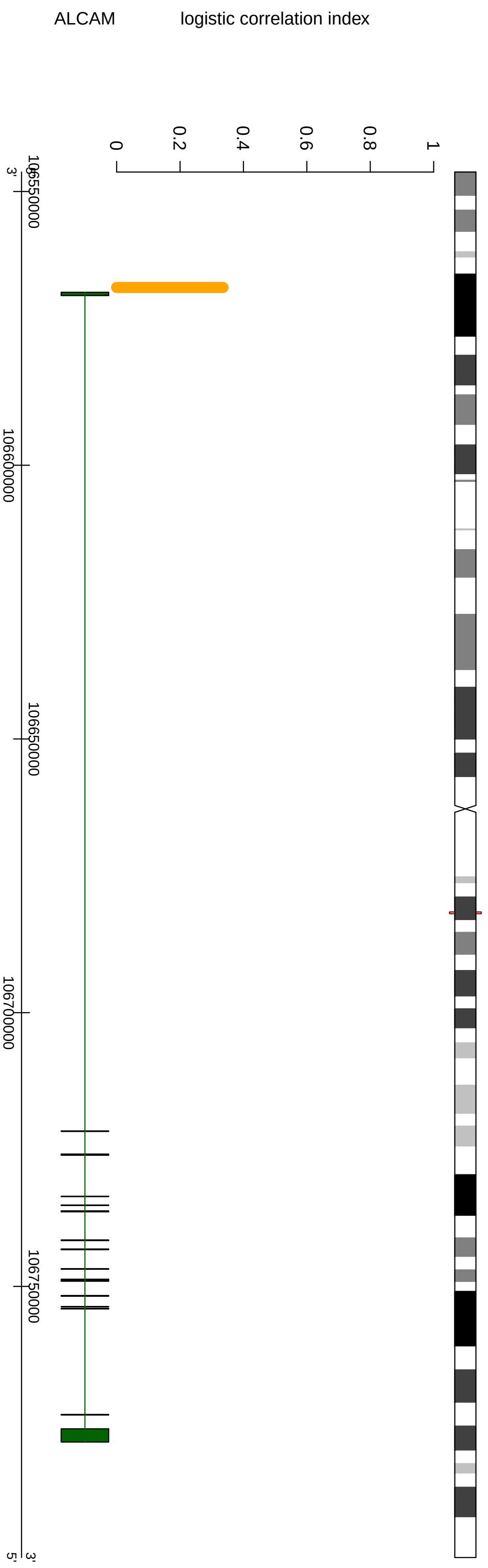
ALCAM
logistic correlation index
0
0.2
0.4
0.6
0.8
1
106550000
106600000
106650000
106700000
106750000
3'
5'
3'

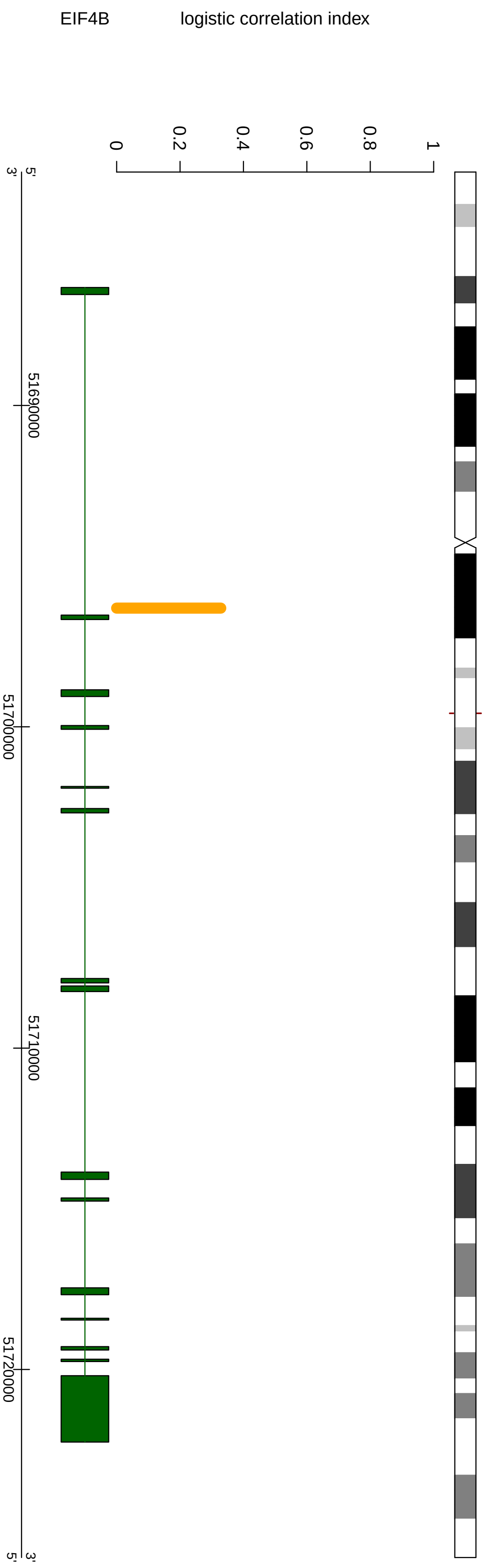
EIF4B
logistic correlation index
0
0.2
0.4
0.6
0.8
1
5'
3'
51690000
51700000
51710000
51720000
3'
5'

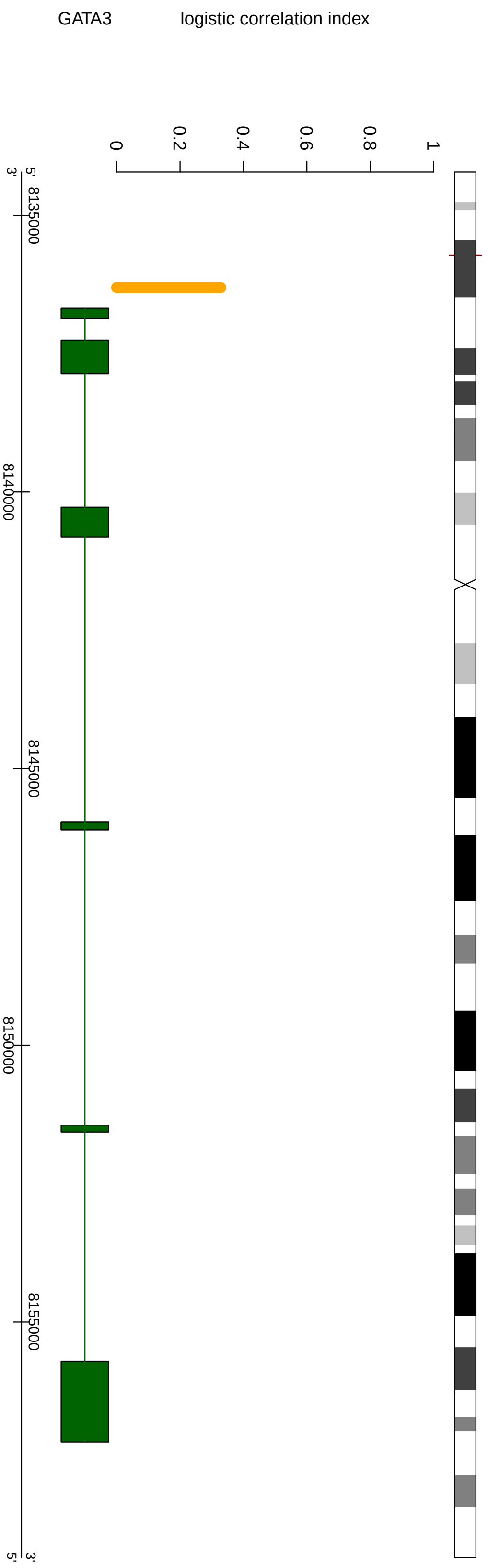
GATA3
logistic correlation index
0
0.2
0.4
0.6
0.8
1
8135000
8140000
8145000
8150000
8155000
5'
3'

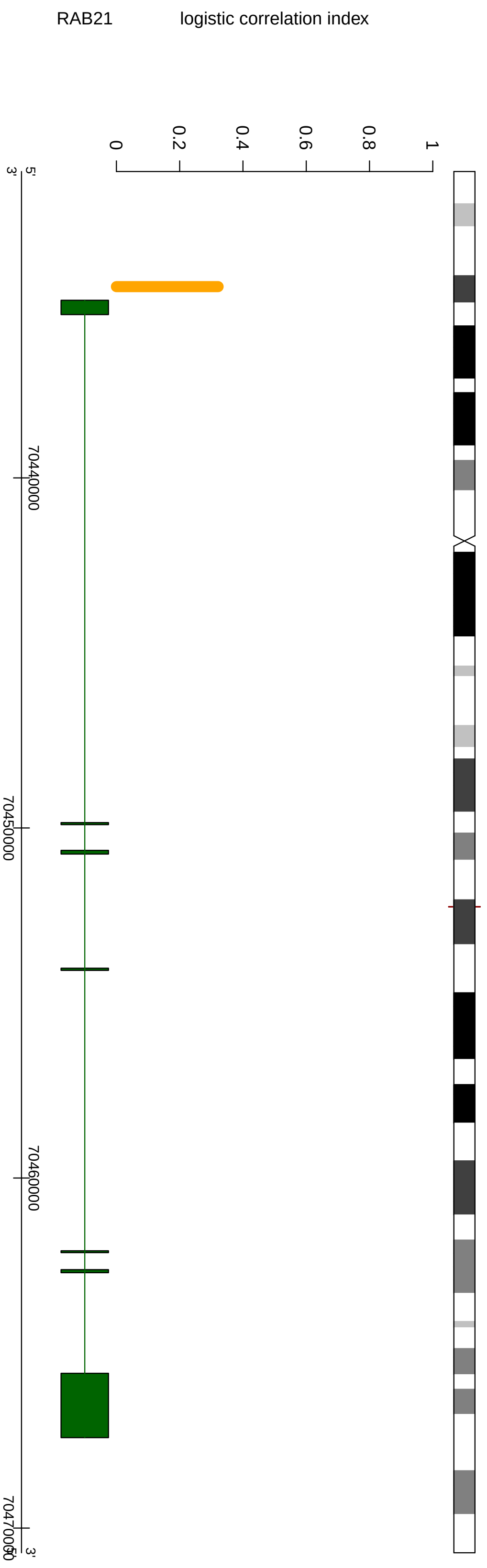
RAB21
logistic correlation index
0
0.2
0.4
0.6
0.8
1
5'
3'
70440000
70450000
70460000
70470000

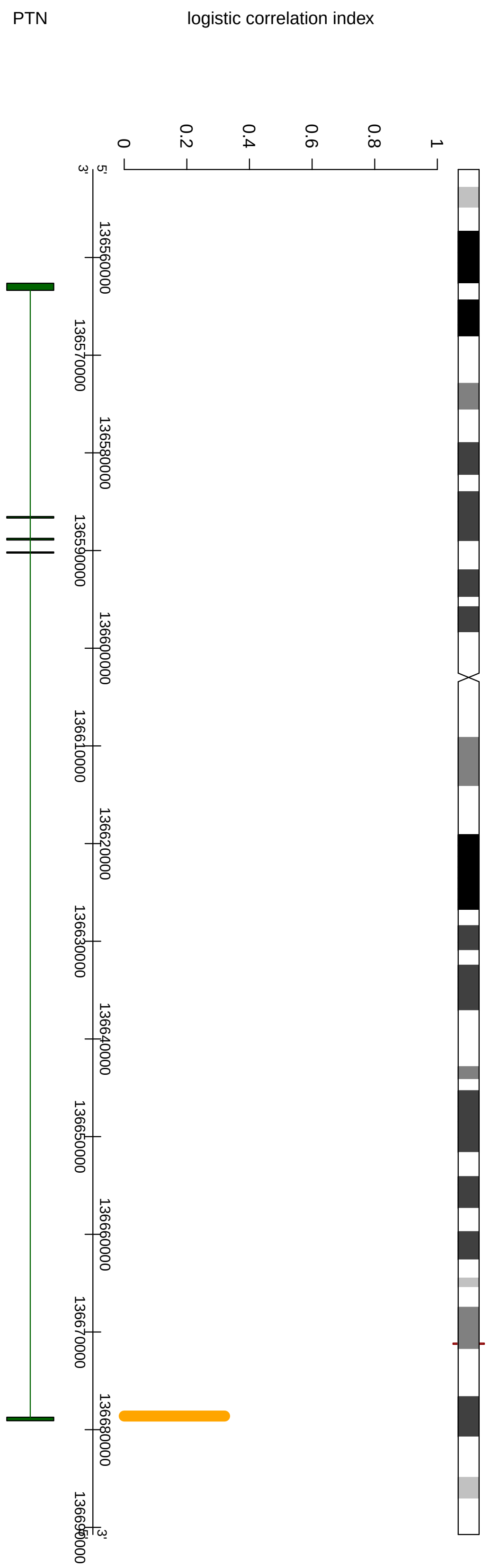
PTN
logistic correlation index
0
0.2
0.4
0.6
0.8
1
5'
3'
136560000
136570000
136580000
136590000
136600000
136610000
136620000
136630000
136640000
136650000
136660000
136670000
136680000
136690000

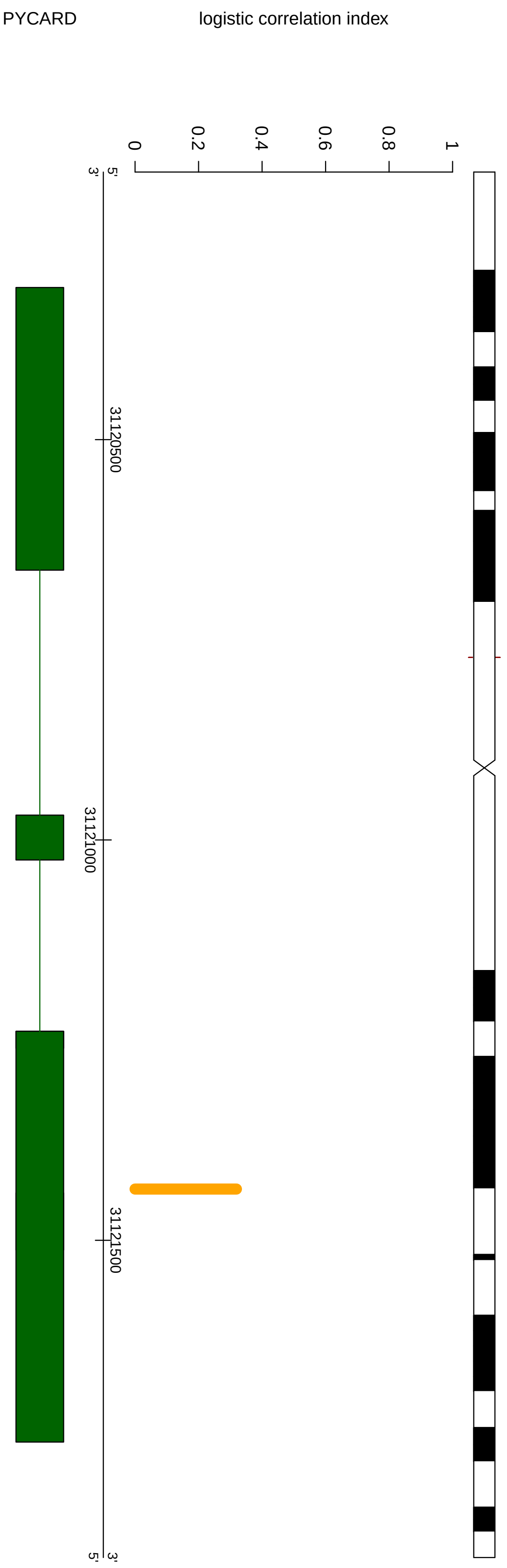
PYCARD
logistic correlation index
0
0.2
0.4
0.6
0.8
1
5'
3'
31120500
31121000
31121500

MAPK13 logistic correlation index

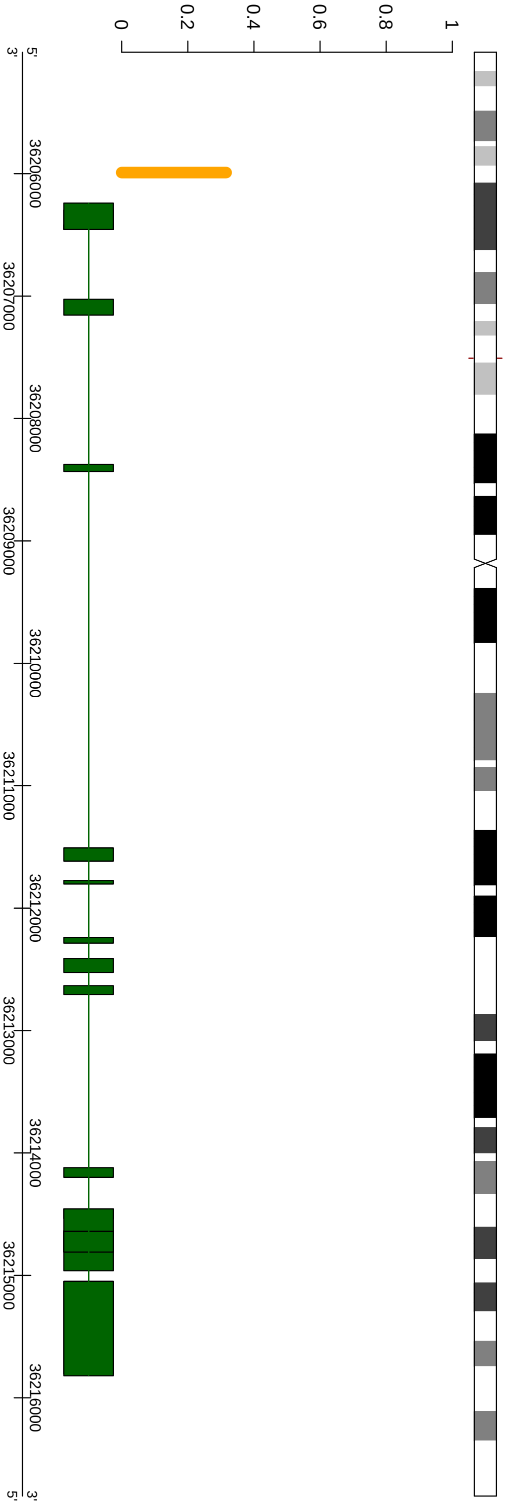

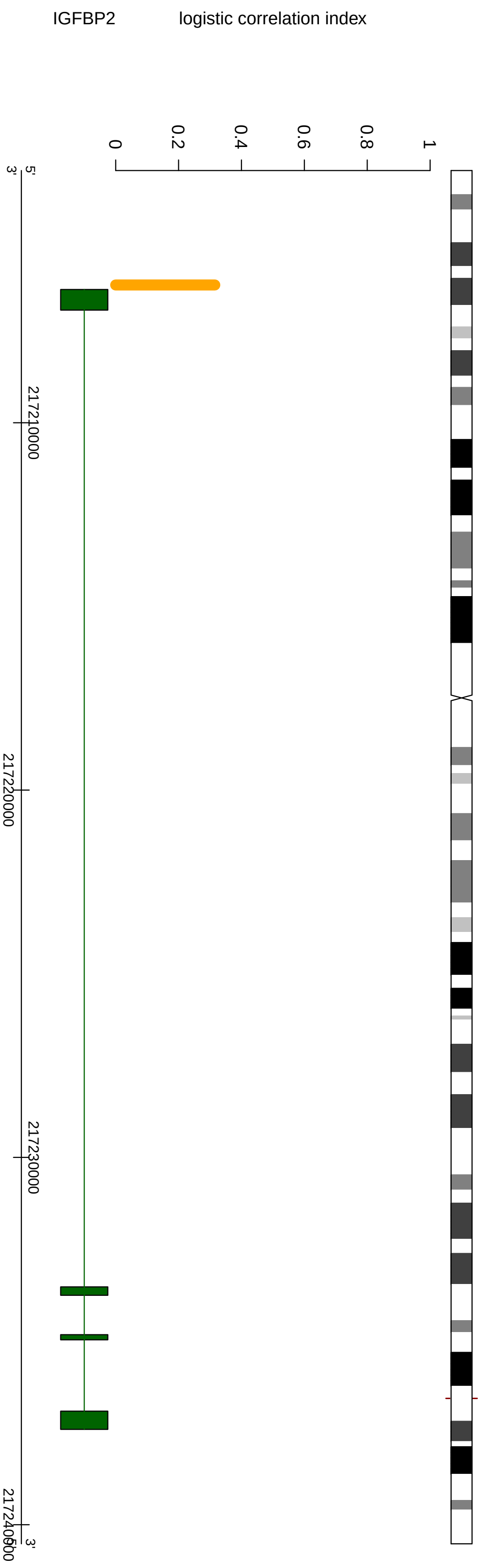
IGFBP2
logistic correlation index
0
0.2
0.4
0.6
0.8
1
5'
3'
217210000
217220000
217230000
217240000
3'
5'

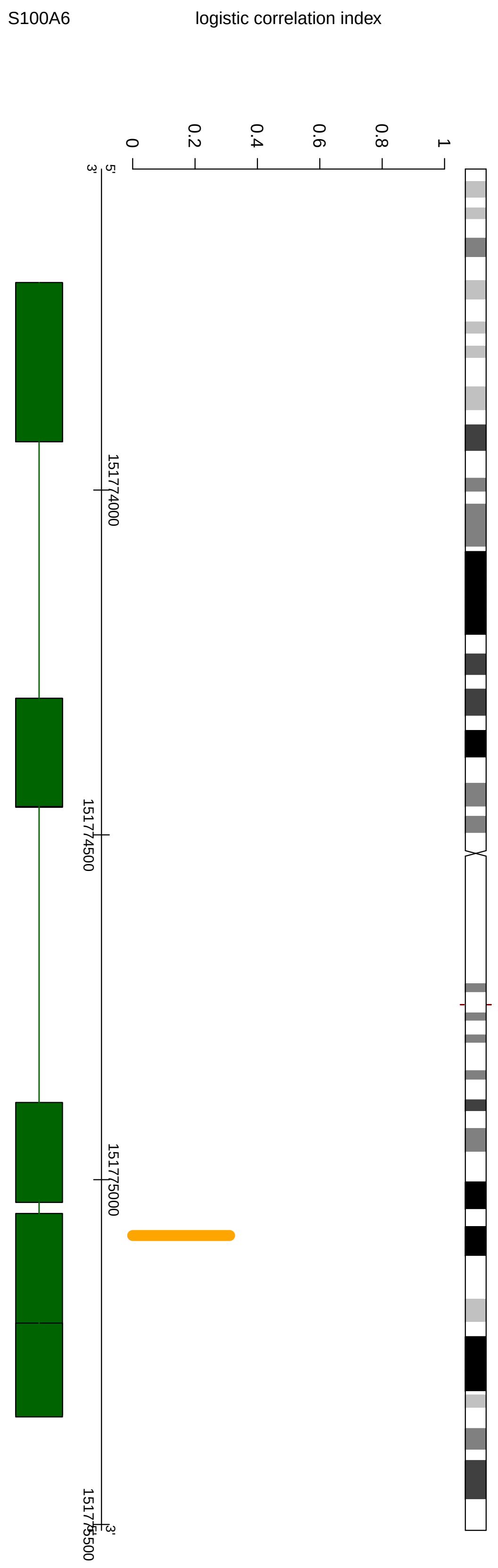
S100A6
logistic correlation index
0
0.2
0.4
0.6
0.8
1
5'
3'
151774000
151774500
151775000
151775500
3'

C12orf24 logistic correlation index

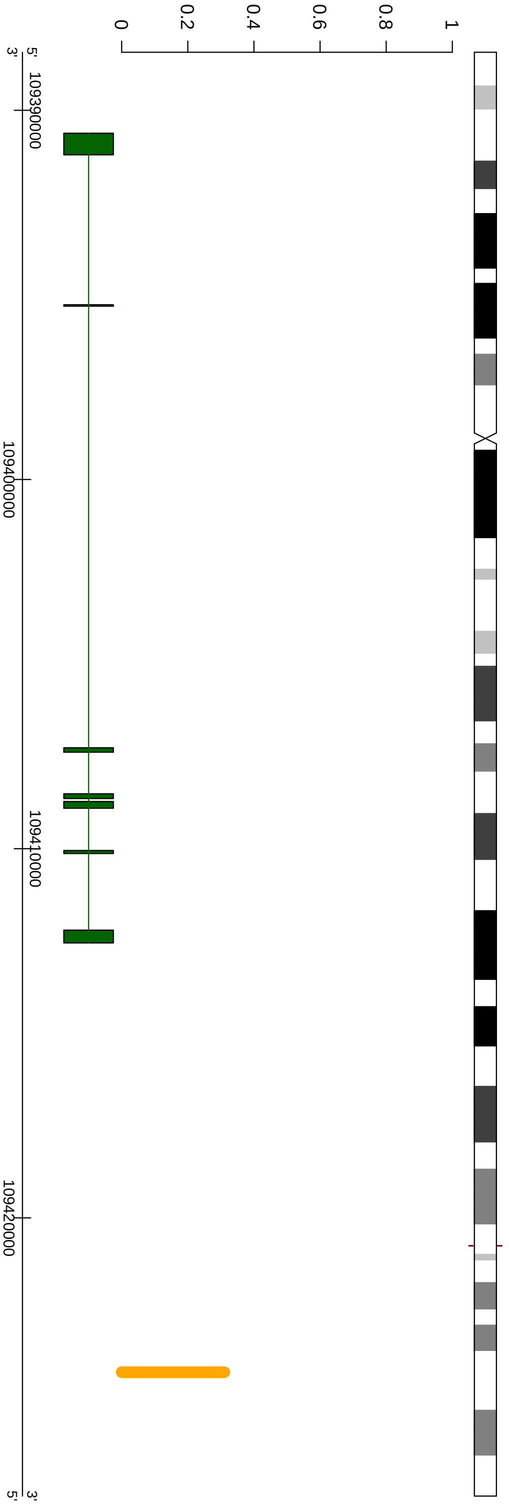

IGFBP7 logistic correlation index

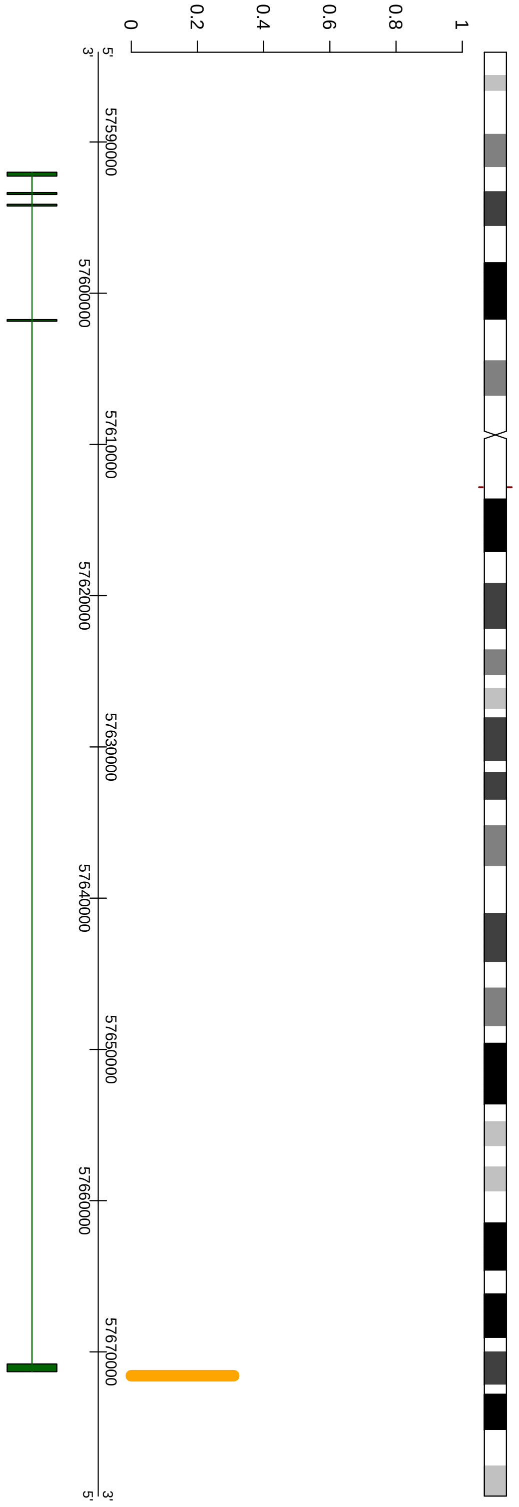

ALDH4A1 logistic correlation index

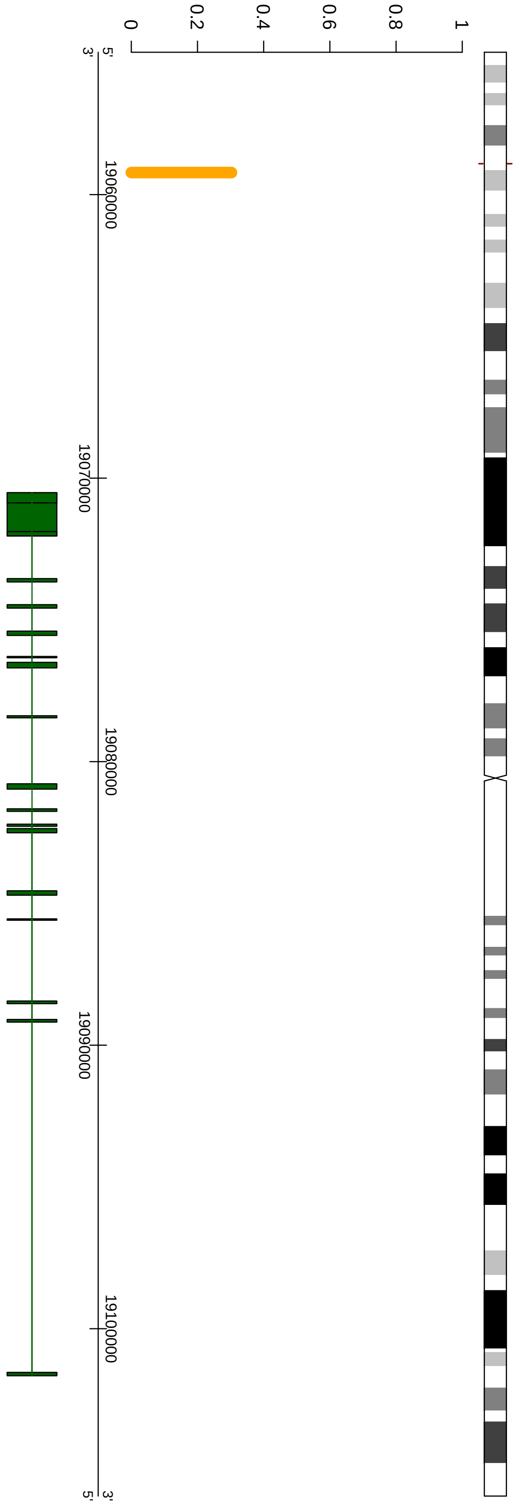

APITD1 logistic correlation index

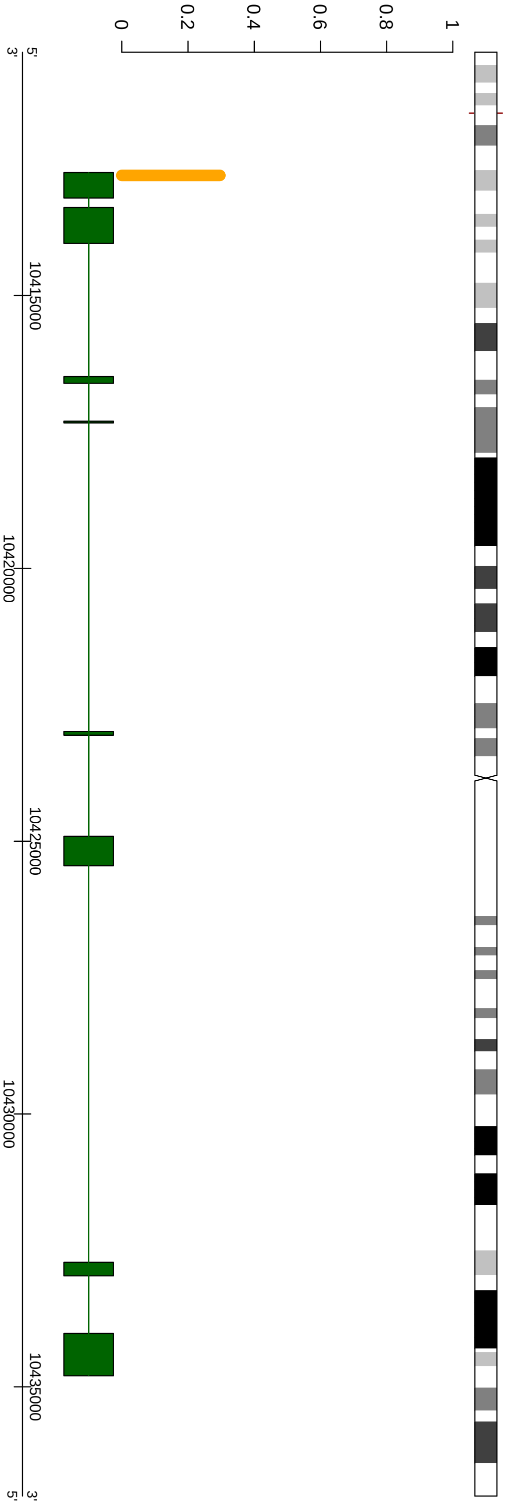

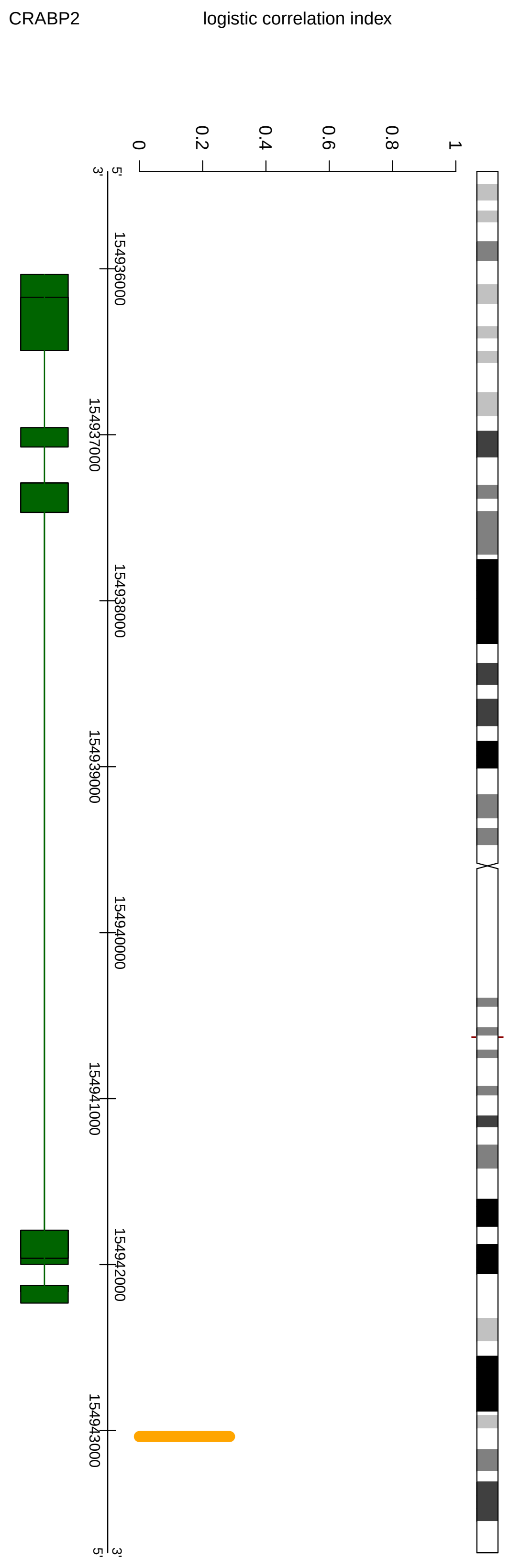
CRABP2
logistic correlation index
0
0.2
0.4
0.6
0.8
1
154936000
154937000
154938000
154939000
154940000
154941000
154942000
154943000
5'
3'

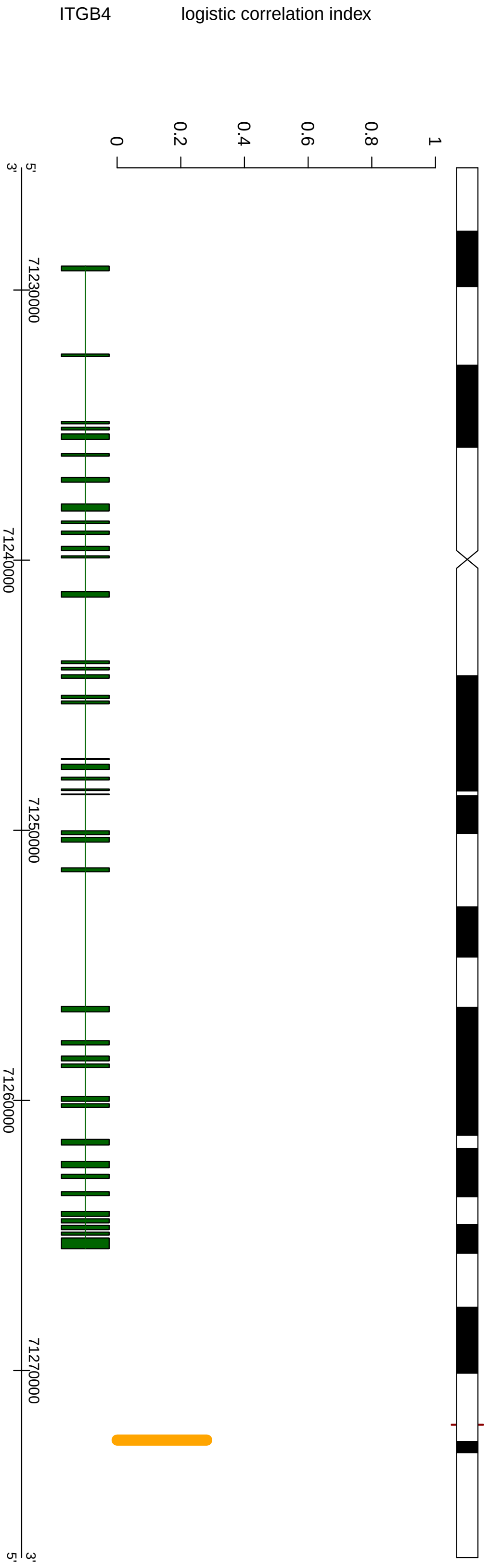
ITGB4
logistic correlation index
0
0.2
0.4
0.6
0.8
1
5'
3'
71230000
71240000
71250000
71260000
71270000
3'
5'

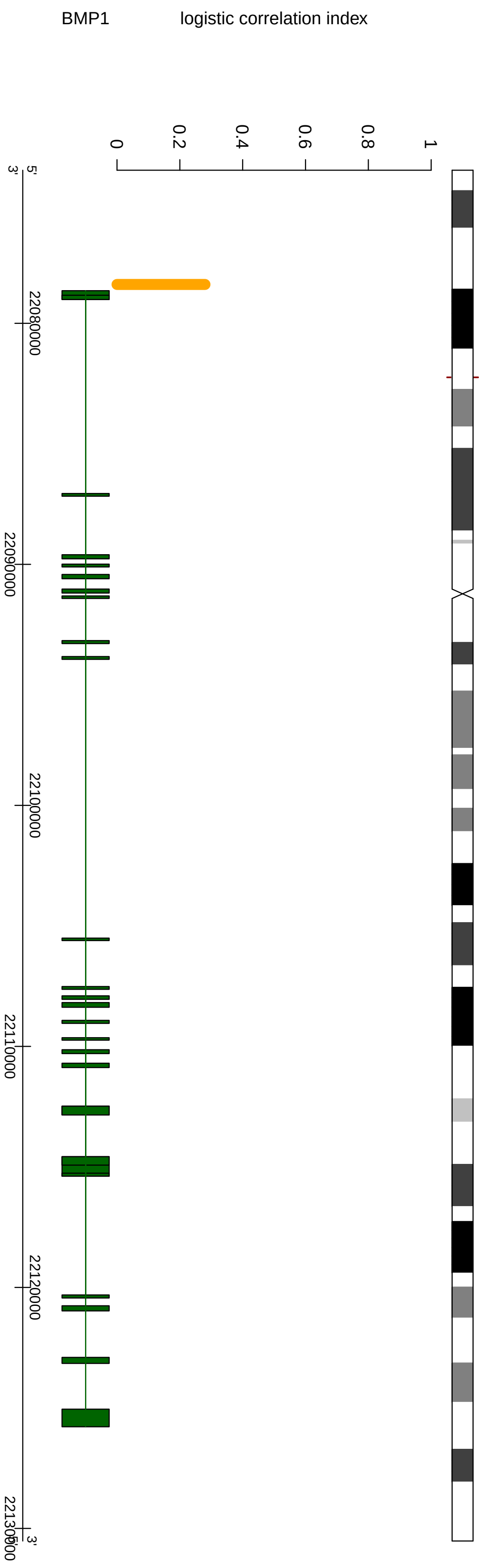
BMP1
logistic correlation index
0
0.2
0.4
0.6
0.8
1
5'
3'
22080000
22090000
22100000
22110000
22120000
22130000
3'

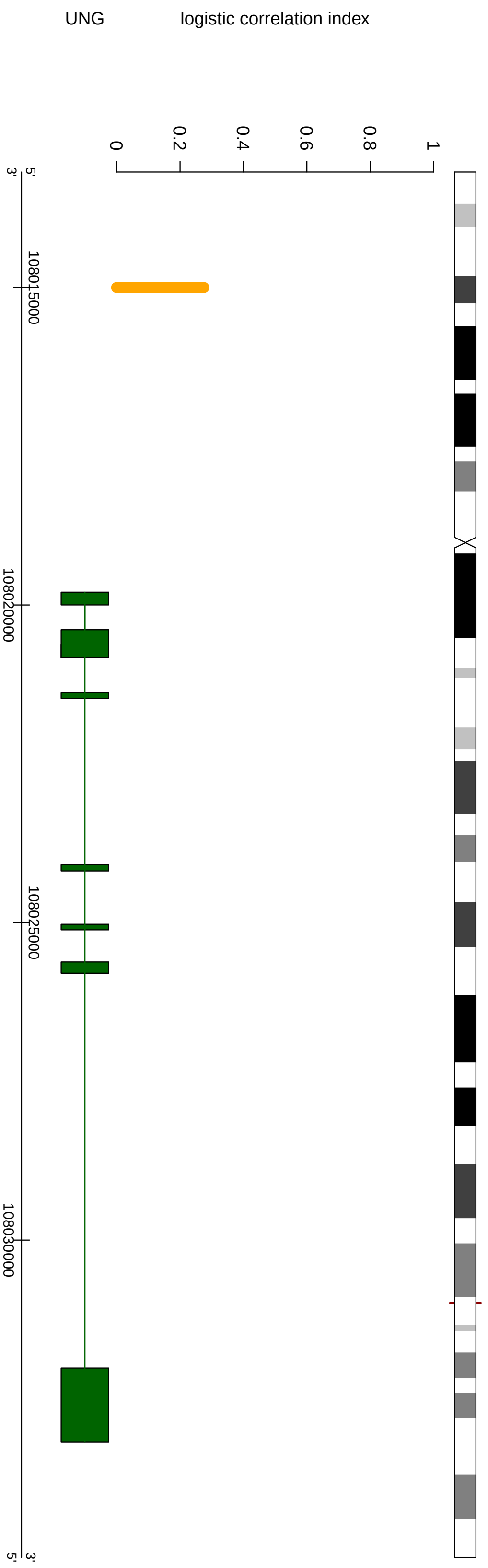
UNG
logistic correlation index
0
0.2
0.4
0.6
0.8
1
5'
3'
108015000
108020000
108025000
108030000
3'
5'

FAM134A logistic correlation index

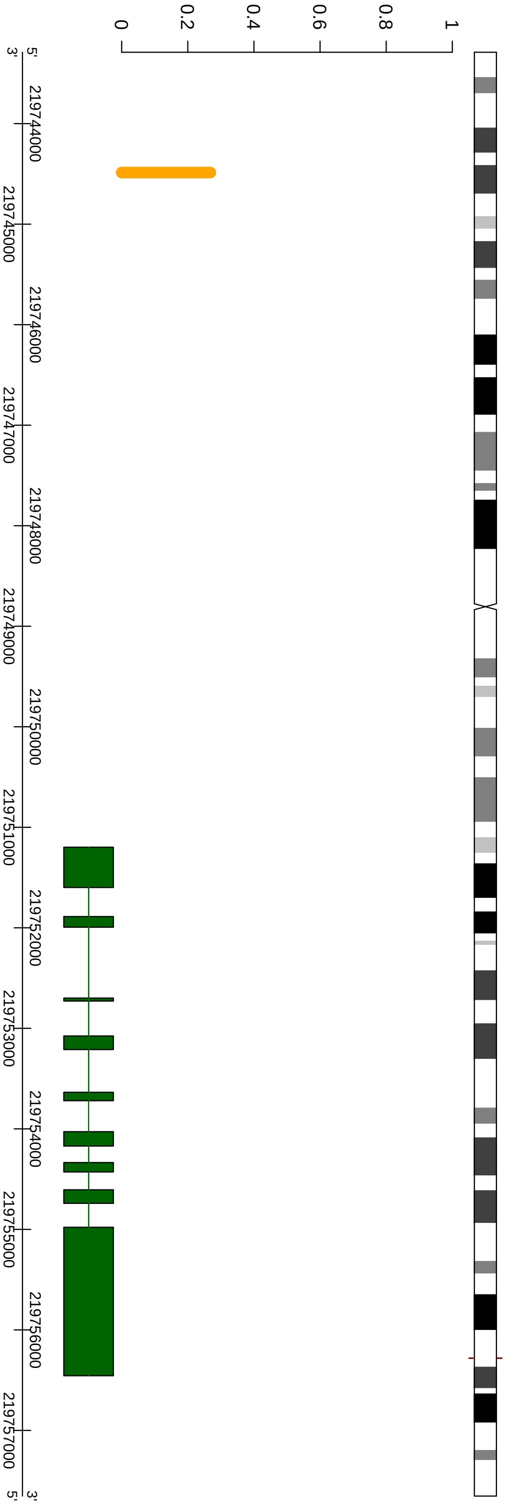

Supplement: Additional file 1 — Maps of methylation for the epigenetically regulated genes. Each graph shows a map of methylation in relation to the top 58 epigenetically regulated genes from Table 2. The respective R values are represented by the magnitude of the yellow bars, plotted over the methylation site responsible for the highest ranked association with each gene [file 1471-2105-11-305-S1.PDF]
